# Chemical, Sensory Variations in Black Teas from Six Tea Cultivars in Jingshan, China

**DOI:** 10.3390/foods14091558

**Published:** 2025-04-29

**Authors:** Rui Wu, Huiling Liang, Nan Hu, Jiajia Lu, Chunfang Li, Desong Tang

**Affiliations:** College of Tea Science and Tea Culture, Zhejiang Agriculture & Forestry University, Hangzhou 311300, China; 13348190817@163.com (R.W.); hlliangtea@icloud.com (H.L.); auster_hoo@163.com (N.H.); challeolu@163.com (J.L.); lichunfang_2005@126.com (C.L.)

**Keywords:** black tea, tea cultivars, color, taste, sensory evaluation, health effects, aroma, molecular docking

## Abstract

The development of black tea quality is the outcome of the synergistic interaction between tea cultivars and the ecological environment of the production area, including factors such as climate, soil, and cultivation practices. Nevertheless, within a specific geographical region, systematic analysis of the environmental regulation mechanisms governing processing adaptability and quality formation among different cultivars remains insufficient. This study evaluated six Camellia sinensis cultivars from the Jingshan region of Hangzhou, China, integrating non-targeted metabolomics, sensory profiling, bioassays, and molecular docking to elucidate cultivar-specific quality attributes. Non-volatile metabolomics identified 84 metabolites linked to color and taste, including amino acids, catechins, flavonoid glycosides, and phenolic acids. Sensory and metabolite correlations revealed that amino acids enhanced brightness and imparted fresh-sweet flavors, while catechins contributed to bitterness and astringency. Specific metabolites, such as 4-hydroxybenzoyl glucose and feruloyl quinic acid, modulated color luminance. Volatile analysis identified 13 aroma-active compounds (OAV ≥ 1), with 1-octen-3-ol, phenylacetaldehyde, and linalool endowing JK with distinct floral-fruity notes. Molecular docking further demonstrated interactions between these volatiles and olfactory receptors (e.g., OR1A1 and OR2J2), providing mechanistic insights into aroma perception. These findings establish a robust link between cultivar-driven metabolic profiles in black tea, offering actionable criteria for cultivar selection and quality optimization in regional tea production.

## 1. Introduction

Tea cultivars are a key determinant of tea quality. Studies have shown that there are significant differences in the metabolite composition of different tea cultivars, which is an important basis for affecting the quality of black tea [1,2]. For example, tea cultivars with high polyphenol content and rich aroma precursors can be converted into more specific flavor compounds during processing [3], thus giving black tea a unique aroma and taste [4]. In addition, even within the same cultivar, differences in the growing environment can lead to significant changes in chemical characteristics [5], indicating that the metabolic characteristics of tea cultivars can vary depending on environmental conditions.

The Jingshan area currently mainly cultivates tea cultivars such as Yingshuang (strong cold resistance), Maov (unique aroma), Cuifeng (good disease resistance), Jiukeng group cultivars (strong adaptability), and Longjing 43# (high yield), which are widely promoted in the region due to their excellent characteristics. Jingshan is a traditional green tea production region. In recent years, it has started to explore black tea production. However, the mechanism underlying the correlation between the sensory qualities, such as aroma, taste, and soup color, of black tea produced from different tea tree varieties in the Jingshan area and their associated metabolites remains unclear. Aroma, taste, and color are important factors in evaluating the sensory quality of tea soup [6]. Since the relationship between the aroma, taste, and soup color of black tea from different tea cultivars in the Jingshan area and related metabolites is still unclear, in-depth research on the differences in the chemical composition and flavor characteristics of black tea from different tea cultivars in the Jingshan area is of great significance for revealing the mechanism of quality formation and enhancing the value of development and utilization.

In this study, taking the Jingshan tea-producing area as an example, fresh leaves of six tea cultivars, namely, Baiye 1#, Longjing 43#, Maolv, Yingshuang, Cuifeng, and Jiukeng, were collected from tea gardens in the same region as the origin of Jingshan tea. The Jingshan black tea sample were prepared using a uniform processing technique (withering, rolling, fermentation, and drying). Through untargeted metabolomics and quantitative analysis of sensory quality, the differences in metabolites and their effects on the sensory quality of black tea made from different tea cultivars were revealed, providing a theoretical basis for the breeding of local tea cultivars and the targeted regulation of black tea quality.

## 2. Materials and Methods

### 2.1. Preparation of Black Tea Samples

Six fresh-leaf tea cultivars, including Baiye 1# (BY), Longjing 43# (LJ), Maolv (ML), Yingshuang (YS), Cuifeng (CF), and Jiukeng (JK), were collected simultaneously in May 2024 from an experimental site located in Jingshan Town, Yuhang District, Hangzhou, Zhejiang Province, China (30°20′ N, 119°50′ E). The region has a subtropical monsoon climate characterized by an average annual temperature of 17.5 °C and annual precipitation of 1500 mm. The soil in the tea garden is yellow-red loam with a pH range of 4.5–5.3, organic matter content exceeding 2.8%, and a terrain gradient of 15–25°. All tea plants were cultivated under identical agricultural practices, and leaf samples were harvested according to a standardized picking criterion of one bud and two leaves.

Upon collection, the fresh leaves were promptly transferred to a controlled-environment withering room (temperature 25 ± 1 °C, relative humidity 70% ± 5%). The leaves were evenly distributed on a stainless steel mesh rack (thickness ≤ 5 cm) to ensure adequate ventilation. Real-time monitoring of moisture content was conducted using a high-frequency impedance moisture analyzer (AND MX-50, AND Corporation, Tokyo, Japan accuracy ± 0.1%). Every two hours, three leaves from each of the six varieties were randomly selected for non-destructive testing, while simultaneous calibration and verification were performed using the 105 °C oven method (GB 5009.3-2016), achieving a high correlation coefficient (R^2^ = 0.993). To maintain consistent water loss across samples, the leaves were manually turned hourly, and the airflow distribution was periodically adjusted by reversing the axial fan’s direction. These measures ensured that the water loss rate difference among varieties did not exceed 5% (ANOVA, *p* > 0.05). All samples achieved the target moisture content (60% ± 1.5%) within 15.5–16.3 h, with final moisture values ranging from 59.8% to 61.5% across the varieties. After that, they were rolled for 1.5 h using a tea rolling machine (6CR-45, Shangyang Machinery Ltd., Hangzhou, China) and then subsequently fermented at 35 °C with 95% relative humidity for 3.5 h in a fermentation machine (JY-6CHF-7, Jiayou Machinery Intelligent Technology Co., Ltd., Quanzhou, China). Finally, drying was performed until complete desiccation using a drying machine (JY-6CHZ-9B, Jiayou Machinery Intelligent Technology Co., Ltd., Quanzhou, China). Biology and technology replicated the methodology described by Long et al. [7]. All black tea samples were manufactured three times using an identical process. Following sampling, the batches were thoroughly blended to obtain a total of 90 g per sample. Each black tea sample was subjected to triplicate sampling, yielding a total sample weight of 270 g. All samples were stored at −40 °C before analysis.

### 2.2. Chemicals and Reagents

Ethyl acetate, n-butanol, sodium bicarbonate, oxalic acid, ethanol, sodium carbonate, sodium hydroxide, Folin-Ciocalteu reagent, anthrone, concentrated sulfuric acid, anhydrous glucose, glutamic acid, potassium dihydrogen phosphate, disodium hydrogen phosphate, and sodium chloride were of analytical grade (>98%). Theanine, caffeine (CAF), (+)-catechin (C), (-)-epicatechin (EC), (-)-gallocatechin (GC), (-)-epigallocatechin (EGC), catechin gallate (CG), (-)-gallocatechin gallate (GCG), EGCG, (-)-epicatechin gallate (ECG), 3-methyl-1-phenyl-2-pyrazolin-5-one (PMP), DPPH radical, 4-nitrophenyl β-D-glucopyranoside, and α-glucosidase were purchased from Shanghai Yuanye Biotechnology Co., Ltd. (Shanghai, China), with a purity higher than 98%. The mixture of normal alkanes (C6-C40) was purchased from Sigma Company in Shanghai, China. Methanol (HPLC grade), propanol (HPLC grade), formic acid (HPLC grade), and acetonitrile (HPLC grade) were purchased from Thermo Fisher Scientific, Inc. (Waltham, MA, USA).

### 2.3. Color Quantitative Analysis

The infusion solutions of black tea samples from six different tea cultivars were prepared following the Chinese Tea Sensory Evaluation Procedure (GB/T 23776-2018 [8]). The color analysis of the tea infusions was conducted using a computer vision system as described by Huang et al. [9]. Specifically, 3.00 g of each tea sample was steeped in 150 mL of boiling water for 5 min in tea bowls, after which the infusion was immediately filtered into another tea bowl. The photographic capture of the infusions was performed using a Nikon Z30 camera (Tokyo, Japan) with the following settings: no flash, 90 mm focal length, ISO 500, manual mode, F/11 aperture, and 1/125 exposure time. The resulting images of the tea leaves and infusions were analyzed using ImageJ software (version 1.51, National Institutes of Health, Bethesda, Bethesda, MD, USA) to determine the color parameters L* (lightness, 0–100), a* (green-red axis), and b* (blue-yellow axis). The chroma (C*ab) was calculated as the Euclidean distance from the color space origin, with higher values indicating more vivid and saturated colors. The hue angle (h◦ab) was determined to represent the dominant wavelength of the color, ranging from 0° (red) to 90° (yellow), 180° (green), 270° (blue), and back to 360° (red). The C*ab and h◦ab values were computed using the following formulas:

C*_ab_ = (a*^2^ + b*^2^)^1/2^

h◦_ab_ = arctan (b*/a*)

### 2.4. Quantitative Analysis of Taste

The taste assessment experiment involved ten trained assessors who evaluated the four key flavor attributes of tea infusion—bitterness, astringency, umami, and sweetness—using a 5-point scale. All taste standard substances were food-grade to ensure participant safety. While no formal human ethics committee approval or documentation process was utilized, appropriate protocols were implemented to protect participants’ rights and privacy. These included voluntary participation, full disclosure of study requirements and risks, informed consent (written or oral), and strict confidentiality of participant data. The assessors determine the taste score of the tea samples based on the concentration of the corresponding standard material. Table 1 details the concentrations of the four standard flavor solutions and their corresponding ratings.

### 2.5. Determination of Non-Volatile Components

#### 2.5.1. Determination of Tea Pigment

The quantitative analysis of thearubigins (TRs), theaflavins (TFs), and theabrownins (TBs) was performed using a systematic analytical method [10]. The procedure involved precise weighing of 3.0 g of tea sample, which was then steeped in 125 mL of boiling water for 10 min followed by filtration. For solution A, 25 mL of the tea extract was mixed with 25 mL of ethyl acetate (analytical grade), shaken for 5 min, and allowed to separate into layers. The ethyl acetate phase (2 mL) was diluted to 25 mL with 95% ethanol. Solution C was prepared by combining 15 mL of the ethyl acetate phase with 15 mL of sodium bicarbonate solution (25%, 2.5 g/100 mL), shaking for 30 s, separating layers, and diluting 4 mL of the upper ethyl acetate phase to 25 mL with 95% ethanol. Solution B was obtained by mixing 25 mL of the tea filtrate with 25 mL of n-butanol (analytical grade), shaking for 3 min, separating layers, and diluting 2 mL of the lower aqueous phase (after adding 2 mL of saturated oxalic acid solution and 6 mL of water) to 25 mL with 95% ethanol. Solution D was prepared by diluting 2 mL of the aqueous phase (after adding 6 mL of distilled water and 2 mL of saturated oxalic acid solution) to 25 mL with 95% ethanol. The absorbance of solutions A, B, C, and D was measured at 380 nm using a spectrophotometer (Agilent) in a 1 cm cuvette (denoted as EA, EB, EC, and ED, respectively), enabling the quantitative analysis of TRs, TFs, and TBs.

#### 2.5.2. Catechins, Caffeine, Theanine

The quantification of catechins and caffeine was performed with minor modifications to the method described in GB/T 8313-2018 [11]. Briefly, 0.2 g of freeze-dried tea powder was mixed with 4 mL of methanol/water solution (70:30, *v*/*v*). The mixture underwent two rounds of ultrasonic treatment (30 min each), followed by two rounds of standing (4 h each). The supernatant was collected and homogeneously mixed. A 1 mL aliquot of the mixture was centrifuged at 6500 rpm for 10 min, and the resulting supernatant was diluted 40 times with 70% methanol and filtered through a 0.45 μm filter membrane prior to HPLC analysis.

The quantification of theanine was performed in accordance with the method stipulated in GB/T 23193-2017 [12]. Briefly, 1.0 g of freeze-dried tea powder was extracted with 100 mL of boiling water for 30 min. Following filtration through a 0.45 μm membrane, the sample was prepared for injection. The HPLC analysis of theanine was conducted using an Agilent 1260 Infinity II system equipped with a C18 column (Agilent, Santa Clara, CA, USA, 959993-902; 250 mm × 4.6 mm, 5 μm, 120 Å), with a detection wavelength of 210 nm. The mobile phase consists of aqueous solution (A) and acetonitrile solution (B).

### 2.6. Non-Targeted Metabolomics

Determined using the method of Yue et al. [13], finely ground plant materials were extracted using an 80% methanol aqueous solution. Data acquisition was performed on a Thermo Scientific Dionex Ultimate 3000 UHPLC system coupled to a Q-Exactive mass spectrometer (Thermo Fisher Scientific, Waltham, CA, USA). An ACQUITY UPLC BEH C18 column (2.1 mm × 100 mm, particle size of 1.7 μm; Waters, Milford, MA, USA) was maintained at 40 °C. The gradient elution program was implemented as follows: 0–2 min, 98% A (water containing 0.1% formic acid) and 2% B (acetonitrile containing 0.1% formic acid); 2–10 min, linear gradient to 25% B; 10–12 min, linear gradient to 98% B; 12–14 min, isocratic at 98% B; 14–16 min, return to initial conditions. The liquid chromatography flow rate was 0.35 mL/min, with an injection volume of 5 μL. The electrospray ionization (ESI) source was operated in positive and negative modes with the following parameters: capillary temperature 320 °C and source voltage 3.6 kV. The sheath gas flow rate was set to 45 arbitrary units in positive mode and 40 arbitrary units in negative mode, while the auxiliary gas flow rate was maintained at 15 arbitrary units in both modes. The mass scanning range was m/z 70–1050, with MS/MS fragmentation performed using a normalized collision energy of 30 eV. For quality control, mixed quality control (QC) samples were prepared by combining aliquots from all individual samples and injected at regular intervals during the LC-MS run. Metabolites were identified based on accurate mass measurement (mass error < 5 ppm) and MS/MS fragmentation patterns.

### 2.7. Analysis of Aromatic Components

The extraction and analysis of tea sample aroma components were performed following the method of Zhang et al. [14] with minor modifications. Specifically, 0.6 g of tea sample and 2 g of sodium chloride were weighed and placed in a 20 mL headspace vial. For this, 5 mL of water and 10 μL of ethyl decanoate solution (10 mg/L) were added as internal standards. A magnetic rotor was included, and the vial was placed in a 40 °C constant-temperature magnetic stirring water bath for 5 min equilibration. The SPME extraction head was then inserted for 45 min adsorption, after which it was removed and inserted into the GC-MS injection port for 5 min desorption at 250 °C.

#### 2.7.1. GC-MS Analytical Conditions

The analysis was performed using a DB-5MS gas chromatography column (30 m × 0.25 mm, 0.25 μm). The temperature program was as follows: an initial temperature of 40 °C held for 5 min, increased to 70 °C at 2 °C/min, further increased to 120 °C at 3 °C/min, and finally raised to 260 °C at 10 °C/min and held for 2 min. Helium (99.99%) served as the carrier gas. The mass spectrometer was operated in positive ion mode with a mass scan range of m/z 30–400, electron energy of 70 eV, and splitless injection method.

#### 2.7.2. Qualitative Analysis

Based on the data acquired through GC-MS analysis, the retention index (RI) of all detected volatile components was calculated using the retention time (RT) of n-alkanes (C6-C40). The compounds were identified by searching the NIST 20 standard spectral library and comparing the RI values with those in the relevant database [15]. Relative content analysis was performed using ethyl decanoate as the internal standard.

#### 2.7.3. Determination of OAV (Odor Activity Value)

OAV_i_ = C_i_/OT_i_, where Ci represents the relative concentration of volatile substances and OT_i_ denotes the olfactory threshold of that volatile substance. Compounds with an OAV > 1 are considered key contributors to the sample’s aroma profile, as they exceed the human detection threshold and significantly influence perceived flavor.

#### 2.7.4. Quantitative Descriptive Analysis (QDA) of Aroma

The sensory evaluation team consisted of 11 assessors (5 male, 6 female) aged 20–33. Team members underwent a three-month olfactory perception training program, which included exposure to various standard samples and foods to enhance sensory discrimination abilities, following the method by Zhang et al. [14] with minor modifications. Evaluations were conducted in a controlled environment maintained at 21 ± 1 °C. Aroma intensity was assessed using a 0–10 scale (0–2: very weak; 2–4: weak; 4–6: moderate; 6–8: strong; and 8–10: extremely strong), with results presented as radar charts. Prior to participation, informed consent was obtained through a statement acknowledging confidentiality and voluntary participation.

#### 2.7.5. Molecular Docking

Molecular docking analysis was performed using AutoDock software (version 4.2.6, Scripps Research, USA). Three-dimensional structural models of characteristic aroma compounds and olfactory receptors (ORs) were obtained from the UniProt database. The following five broad-spectrum olfactory receptors were selected as research subjects: OR1A1 (UniProt ID: Q9P1Q5), OR1G1 (UniProt ID: P47890), OR2W1 (UniProt ID: Q9Y3N9), OR1D2 (UniProt ID: P34982), and OR52D1 (UniProt ID: Q9H346). Five key aroma compounds from the JK samples—1-Octen-3-ol, 1-Heptanol, Phenylacetaldehyde, β-Ocimene, and linalool—were chosen as ligands to investigate their interactions with the aforementioned olfactory receptor proteins. The structure of the human olfactory receptor protein was downloaded in PDB format from AlphaFold (version v2.2.0). The ORs were dehydrated hydrogenated and small molecules were hydrogenated, and finally exported to PDBQT format files. The predicted binding pocket was used to generate the most rational conformation through docking selection. The ligand-OR conformation file was imported into LigPlot+ software (version 2.2.9) to generate a 2D interaction diagram and analyze the binding mechanism. Finally, the 3D binding site interaction diagram between the aroma substances and amino acid residues was plotted using PyMOL software (version 3.1.4, DeLano Scientific LLC, USA).

### 2.8. Statistical Analysis

Three independent replicates were performed per experimental group, and data were expressed as mean ± standard deviation (SD). Statistical significance was assessed via one-way ANOVA with Duncan’s multiple range test using SPSS (version 30.0.0, IBM, USA). A threshold of *p* < 0.05 was applied to determine significant differences. The metabolic profile comparisons were analyzed using PCA and OPLS-DA via SIMCA 14.1 software. PCA projects samples onto a reduced-dimensional space to enhance data visualization by capturing the first few principal components. In contrast, OPLS-DA, a supervised method, maximizes class separation by emphasizing influential discriminatory metabolites across sample grades. A permutation test validated the OPLS-DA model and assessed overfitting risk. The R^2^ value indicates model fit, while Q^2^ evaluates predictive power [16,17]. The dataset was not pre-processed. For PCA, variables were scaled to unit variance without transformation. For OPLS-DA, a Parafac-based multilinear scaling method was used to handle multidimensional datasets [18,19].

## 3. Results and Discussions

### 3.1. The Effects of Tea Cultivars on Color and Taste Characteristics of Black Teas

#### 3.1.1. Colorimetric Values and Taste Score

**Figure 1:**
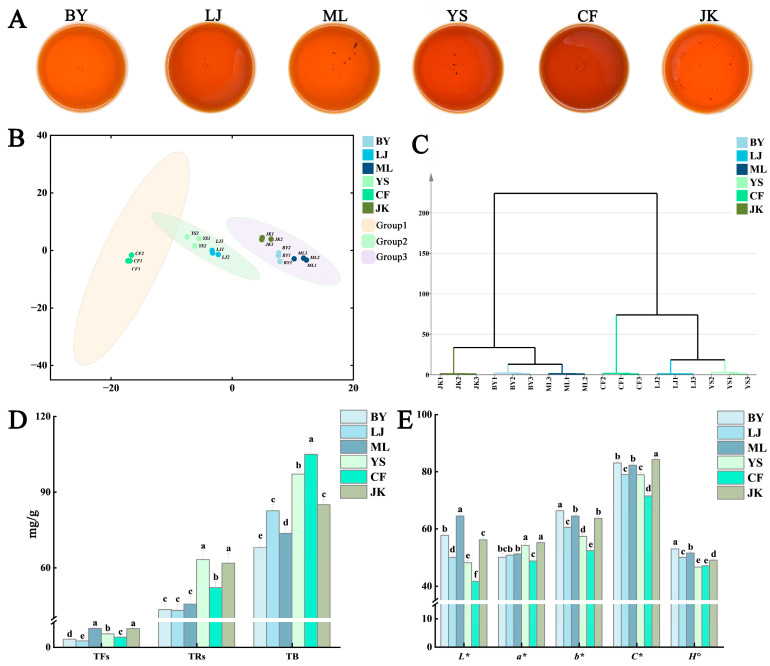
Red tea color, multivariate statistical analysis, and tea pigment content of different tea cultivars: (**A**) photography of tea soup; (**B**) PCA analysis of colorimetric values; (**C**) HCA analysis of colorimetric values; (**D**) tea pigment content; and (**E**) colorimetric values. Note: Different letters indicate a statistically significant (*p* < 0.05) difference between groups.

Tea soup images were captured under controlled conditions (Figure 1A), followed by quantitative analysis of the Lab and LCH colorimetric systems for Jingshan black tea soups from six tea cultivars (Figure 1E). Results demonstrated significant color differences among cultivars. As shown in Figure 1B,C, color difference analysis combined with principal component analysis (PCA) revealed distinct grouping patterns among the six tea cultivars. The PCA plot demonstrated significant separation of the samples into three distinct clusters: one comprising the CF samples, another including YS and LJ samples, and a third consisting of ML, BY, and JK samples. This result aligns with the separation patterns observed in hierarchical cluster analysis (HCA). The CF sample demonstrated the lowest luminance value (L*), with significantly reduced red-green chroma (a*), yellow-blue chroma (b*), and color saturation (C*) compared to other samples. Conversely, the ML, BY, and JK samples exhibited significantly higher L*, b*, and C* values than the remaining samples.

**Figure 2:**
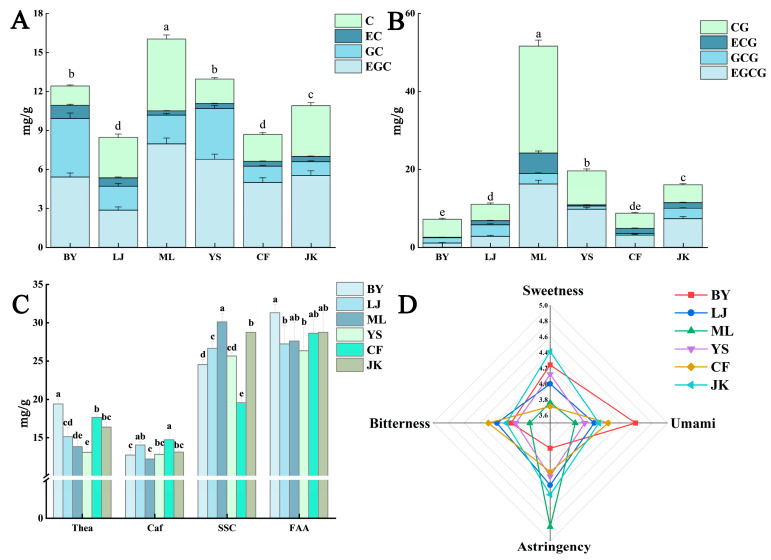
Main non-volatile components and taste radar chart of different tea plant varieties: (**A**) non-ester-type catechin content; (**B**) esterified catechin content; (**C**) theanine, caffeine, soluble sugars, and total free amino acids; and (**D**) taste score radar chart. Note: Different letters indicate a statistically significant (*p* < 0.05) difference between groups.

The umami, sweetness, astringency, and bitterness of all black tea infusions were evaluated through quantitative descriptive analysis using a structured 10-point scale (Figure 2D). Results demonstrated significant differences in taste characteristics among the six tea cultivars. The JK and BY samples exhibited higher sweetness scores, with the BY sample showing the highest umami intensity, followed by the JK sample. The ML sample was distinguished by its pronounced astringency, while the CF sample displayed the most intense bitterness. Overall, umami and sweetness demonstrated a positive correlation, whereas high-astringency cultivars frequently exhibited variability in bitterness intensity. These patterns reflect metabolic differences in polyphenol and alkaloid biosynthesis among the cultivars.

#### 3.1.2. The Principal Non-Volatile Components in Tea Leaves

##### Catechins, Caffeine, Soluble Sugars, and Amino Acids

As shown in Figure 2A–C, the primary non-volatile constituents of black tea samples from six distinct tea cultivars were analyzed, including four ester-type catechins, four non-ester-type catechins, caffeine, soluble sugars, theanine, and free amino acids. Catechins are recognized as the primary source of bitter and astringent flavors in tea infusions. Research has shown that non-ester-type catechins exhibit relatively mild bitter and astringent properties while still contributing significantly to the overall taste and astringency profile; in contrast, ester-type catechins display more intense bitter and astringent characteristics [20]. Amino acids significantly influence tea’s flavor profile, particularly in umami and sweetness expression, while also contributing to aroma and color development during processing [21]. Caffeine is regarded as being associated with bitterness and is a significant constituent contributing to the bitter perception in tea soup [22]. Soluble sugars constitute the primary source of sweetness in tea infusions, mitigating bitter and astringent notes while imparting a mellow character [23]. Theanine, a signature amino acid in tea, comprises over half of the total free amino acids and synergizes with other amino acids to create complex flavor dimensions and a refreshing quality in the infusion [24]. The taste profile differences among these cultivars were correlated with sensory evaluation data presented in radar charts (Figure 2D).

In this experiment, the ML sample exhibited the highest levels of ester-type catechins, particularly EGCG and ECG, which likely contributed to its significantly higher astringency score compared to other varieties. Despite this, it displayed the lowest bitterness, potentially due to the following two factors: the high content of non-ester-type catechin C, which may buffer the bitter sensation, and the significantly higher soluble sugar content compared to other samples, which could neutralize some bitterness from catechins.

The BY sample contained relatively high levels of non-ester-type catechins and the lowest levels of ester-type catechins. Additionally, it had significantly higher levels of theanine and free amino acids than other samples, which likely contributed to its pronounced sweetness and umami flavor. The LJ sample showed relatively low levels of theanine and free amino acids but a high caffeine content. The CF sample had markedly higher caffeine content than other samples, which correlated positively with its bitterness and astringency in sensory evaluations. The JK sample contained relatively high levels of non-ester-type catechins and soluble sugar; this result is consistent with the findings of Zhang et al. [25] on the Jiukeng group cultivars, which might explain its relatively high sensory sweetness value.

##### Tea Pigments

Long et al. [7] established a significant correlation between the color difference value of tea soup and the contents and ratios of theaflavins (TF), thearubigins (TR), and theabrownins (TB). As illustrated in Figure 1D, the CF sample exhibited significantly higher TB content than the remaining samples, while the BY and ML samples demonstrated lower TB content. These samples showed significantly lower and higher L* values, respectively, compared to other samples. This indicates a negative correlation between the L* value of tea soup and TB content, a finding consistent with the research results of Yang et al. [26]. These findings align with previous research. During tea processing, catechin oxidation leads to the formation of TF and TR [27], which significantly influence the color of tea infusions. Zhao et al. [28] demonstrated that TF contributes to a bright red color, while TR imparts a reddish-brown hue. The concentrations of these pigments show positive correlations with the a* and b* values of tea infusions. Specifically, TF and TR exhibit significant positive correlations with L* and a* values, but not with b* values. These findings confirm that pigment content positively correlates with a* and b* values in tea infusions. In this study, TF and TR exhibited significant positive correlations with L* and a* values but not with b* values, presenting a slight discrepancy from previous findings. This divergence may stem from variations in TB content or other non-volatile constituents, warranting further investigation.

#### 3.1.3. The Multivariate Analysis Results of LC-MS-Based Metabolomics

##### Non-Volatile Differential Metabolites

**Figure 3:**
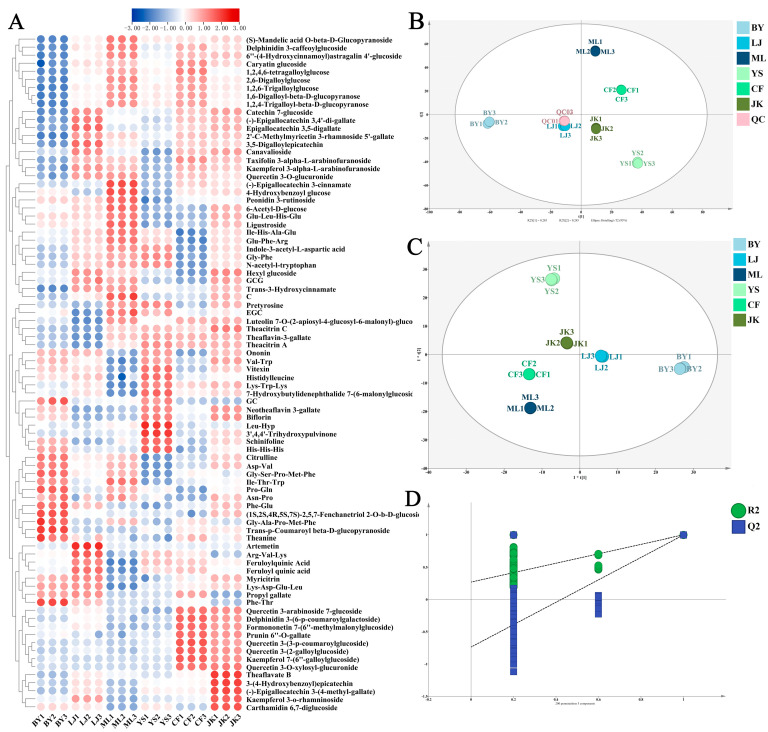
Multivariate statistical analysis of non-targeted differential metabolites in different tea cultivars: (**A**) heatmap of differential metabolites, (**B**) PCA analysis, (**C**) OPLS-DA analysis, and (**D**) permutation test.

To further investigate the color and taste metabolites in black tea infusion, multivariate statistical analysis was performed using UHPLC-Q-TOF-MS data. After preprocessing, 3261 metabolites were detected across six tea cultivars. As shown in Figure 3B, PCA analysis and QC samples validated the repeatability and stability of sample preparation and instrumental analysis. The PCA scatter plot demonstrates tight clustering of QC samples near the coordinate origin, indicating satisfactory instrument repeatability and data reliability for metabolomics analysis. Additionally, an OPLS-DA model (Figure 3C) was constructed to analyze chemical characteristics among different tea cultivars, with parameters showing R^2^X = 0.970 and Q^2^ = 0.999 (Figure 3A), indicating strong explanatory and predictive capabilities. These metrics demonstrate that the model exhibits strong explanatory power and predictive accuracy, effectively distinguishing chemical characteristic variations among different tea cultivars. To further validate the model’s stability, 200 permutation tests were performed (Figure 3D), with results showing no evidence of overfitting. This indicates that the model, using detected non-volatile substances as independent variables to differentiate and predict black tea from various tea cultivars, demonstrates robust stability. The VIP value serves as a critical metric in orthogonal partial least squares discriminant analysis (OPLS-DA) for evaluating variable contributions to model discrimination. Variables with VIP values exceeding 1 are generally considered to significantly contribute to class differentiation. In combination with one-way analysis of variance (ANOVA) followed by Duncan’s test, the significant differences of these differential substances among tea cultivars were further confirmed. Based on criteria of VIP > 1 and ANOVA results (*p* < 0.05), 406 differential substances were identified.

##### Differential Metabolites Related to the Color and Taste of Tea Soups

The supervised OPLS regression model was utilized to assess the significance of metabolites associated with color and taste. In the color OPLS model, absorbance values (L*, a*, b*, C*, and H°) were designated as Y variables, while in the taste OPLS model, taste scores (umami, sweetness, astringency, and bitterness) served as Y variables. The Q^2^ values of the color and taste OPLS regression models were 0.96 and 0.99, respectively, indicating the predictability of pure color and taste values. Permutation test results for both regression models showed that in the validated models, all R² values were below 0.4 and all Q^2^ values were below 0.05. These results confirmed the stability and applicability of the models.

**Figure 4:**
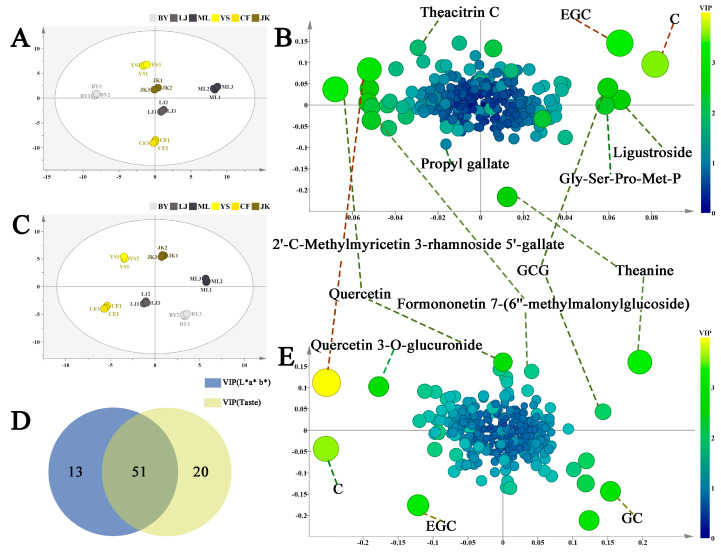
OPLS regression models of the color and taste of black tea soup from different tea cultivars: (**A**) scatter plot of color; (**B**) loading plot of the color OPLS model; (**C**) scatter plot of taste; (**D**) Venn diagram of related differential metabolites; and (**E**) loading plot of the taste OPLS model.

The scatter plots of the color and taste models (Figure 4A,B) reveal distinct clustering patterns among black tea samples from six tea cultivars based on their color and taste characteristics. Moreover, supervised OPLS model loading plots (Figure 4B,E) were used to identify key metabolites differentiating these characteristics. In these plots, metabolite size and color represent VIP values across the OPLS model. Metabolites including epicatechin (EC), epigallocatechin (EGC), quercetin 3-O-glucuronide, 2′-C-methylmyricetin-3-rhamnoside-5′-galloyl ester, and theanine exhibited significantly higher VIP values than other detected substances. These metabolites were identified as crucial differential substances influencing the color and taste characteristics of black teas from different cultivars. These findings suggest that the differential substances among the six types of black tea are implicated in determining the color and taste of black tea. Following VIP value threshold screening (VIP > 1), 84 key differential metabolites associated with color and taste were selected, and a heatmap was plotted (Figure 3A). As shown in Venn diagram 4D, among the identified differential metabolites, 13 compounds were exclusively associated with color characteristics, 21 compounds specifically influenced taste properties, and 51 compounds exhibited significant expression differences in both color and taste models. These findings indicate that metabolite groups affecting tea infusion’s color and taste demonstrate both functional overlaps, such as polyphenol oxidation products influencing both color and taste. These substances likely synergistically regulate black tea quality formation. Table 2 lists the VIP values of key metabolites in the color and taste OPLS models. Results suggest that metabolite differences in black teas from different tea cultivars may be the crucial chemical basis for sensory quality variations.

#### 3.1.4. Correlation and Regression Analysis of Metabolites Associated with the Color and Taste of Tea Soups

**Figure 5:**
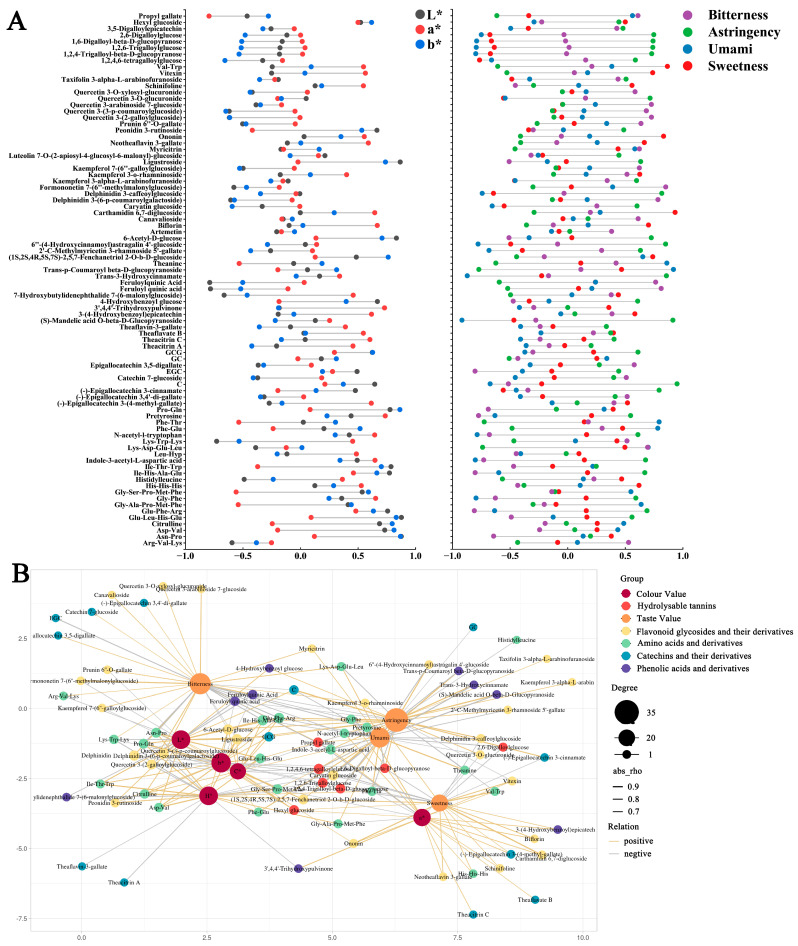
Correlation and regression analysis of metabolites related to the color and taste of tea infusions from different tea cultivars: (**A**) regression coefficients of 84 key compounds related to color and taste in the OPLS regression model and (**B**) correlation analysis of metabolites related to the color and taste of tea infusions from different tea cultivars with colorimetric values and taste scores.

To analyze the dependency relationships between individual metabolites and the color and taste of black tea samples from six different tea cultivars and to explore correlations among all metabolites, a correlation network diagram of metabolites, color, and taste was generated (Figure 5B). Visualizations of metabolite–metabolite correlations and metabolite–color/taste relationships with strong positive correlations (r ≥ 0.7) and strong negative correlations (r ≤ −0.7) are presented in Figure 5A. The font size of each metabolite was determined by the number of significantly correlated metabolites. Metabolites with higher positive regression coefficients may enhance specific color or taste characteristics of the tea infusion, while negative coefficients may indicate inhibitory effects. This method quantitatively assessed each metabolite’s contribution to the tea’s sensory quality. Results demonstrated strong correlations among metabolite contents, colorimetric values, and taste scores.

To validate the predictive capability of the 84 key metabolites for tea infusion color and taste, two orthogonal partial least squares discriminant analysis (OPLS) regression models were reestablished using the metabolites’ mass data. In these models, metabolite mass data served as independent variables (X variables), while tea infusion color parameters (colorimetric values) and taste scores were integrated as dependent variables (Y variables). The model fitting results showed Q^2^ values of 0.961 for color and 0.997 for taste, indicating excellent predictive ability of these key metabolites for both color and taste characteristics.

##### Amino Acids and Their Derivatives

Regarding tea infusion color, amino acids and their derivatives generally show positive correlations with lightness (L*), but have divergent effects on redness (a*) and yellowness (b*). For instance, theanine significantly suppresses redness (r = −0.53), while proline-glutamine dipeptide (Pro-Gln) enhances light transmittance by increasing lightness (r = 0.78). In terms of tea infusion taste, most amino acids and their derivatives generally show positive correlations with fresh and sweet flavors, though some demonstrate negative correlations. For instance, histidine (His) and its derivatives, valine-tryptophan (Val-Trp), and proline-glutamine dipeptide (Pro-Gln) exhibit significant positive correlations with sweetness enhancement. Umami taste is primarily dominated by theanine, which displays a strong positive correlation. While research indicates that theanine’s taste threshold is relatively high and it does not directly influence human perception of umami in tea infusions, it enhances the response to glutamate by activating umami receptors (T1R1/T1R3) [29], and simultaneously inhibits bitter taste receptor signals (hTAS2R16) [30], creating a dual regulatory effect of “umami enhancement-bitterness inhibition”. Bitter and astringent sensations correlate with substances like glutamic acid-phenylalanine-arginine (Glu-Phe-Arg) (r = 0.69), while phenylalanine-threonine (Phe-Thr) shows negative regulation (r = −0.72).

##### Catechins and Their Derivatives

Catechin derivatives, formed through esterification, glycosylation, and oxidative polymerization, are significant contributors to black tea’s flavor, imparting bitter and astringent taste profiles [31]. Their structural and functional diversity considerably impacts the tea’s soup color and taste characteristics. In recent years, the sources of the bitter and astringent tastes of catechins have been widely reported. For example, EGC and ECG have been shown to have a pronounced bitter taste [32].

Regarding tea infusion color, theaflavin C, theaflavin A, and (-)-epigallocatechin 3-(4-methylgalloyl) ester are key regulators of the red hue associated with the a* value (r = 0.6, 0.46, and 0.62). The JK sample’s highest theaflavin C content likely explains its significantly elevated a* value compared to other samples. GC and GCG demonstrate strong positive correlations with the b* value (r = 0.32, 0.63), with the ML sample’s relatively high GCG content potentially contributing to its prominent b* value.

Most catechins and their derivatives exhibit positive correlations with astringency and bitterness. The YS sample’s elevated levels of (-)-epigallocatechin 3-cinnamate likely contribute to its significantly higher astringency score. Theaflavin A enhances bitterness through activation of bitter taste receptors [33], potentially explaining the CF sample’s highest bitterness score. Unlike most catechins and their derivatives, GC exhibits positive correlations with the umami (r = 0.26) and sweet (r = 0.34) tastes of tea soup. This may be attributed to the following two factors: its relatively high threshold for bitter and astringent flavors and its ability to form hydrogen bonds with umami amino acids. This interaction facilitates binding to umami receptors (T1R1/T1R3), thereby enhancing the perceived intensity of umami taste [34]. The significantly higher GC content in the BY sample compared to other samples may be a contributing factor to its exceptionally high umami score. Theaflavin B exhibits a positive correlation with sweetness perception (r = 0.4), potentially through chemical transformations that reduce bitter compounds while enhancing sweet notes. Wei et al. [35] demonstrated that during the yellowing process of tea leaves, which increases theaflavin content, sweetness also increases. As one of the theaflavins, theaflavin B reaches its highest concentration in the JK sample, which may contribute to this sample’s highest sweetness score through modification processes.

##### Phenolic Acids

A total of nine phenolic acid compounds were identified, primarily formed through esterification, glycosylation, and hydroxyl modification. Many previous studies have pointed out that organic acids containing acidic groups affect the brightness value by reducing the pH value [36]. Regarding tea infusion color, 4-hydroxybenzoyl glucose increases molecular polarity via glycosidic bonds, promoting the dispersion of colloidal particles (size < 300 nm) [37,38], which likely explains its positive correlation with the L* value (r = 0.67) and enhanced brightness. The YS sample contained the highest level of 4-hydroxybenzoyl glucose, which may significantly influence the L* value. Feruloylquinic acid demonstrated a negative correlation with the L* value (r = −0.79). While no literature directly references this compound, its ester group may form large-particle colloids (>500 nm) through hydrophobic aggregation [39], potentially reducing the L* value. The CF sample exhibited the highest feruloylquinic acid content, which may contribute to its significantly lower L* value compared to other samples.

##### Flavonoid Glycosides and Their Derivatives

In previous studies, the astringent threshold of flavonoid glycosides was lower than that of catechins, and they had an inhibitory effect on taste qualities such as sweetness [40]. Flavonoid glycosides and their derivatives, formed through glycosidic bonds between flavonoid nuclei and sugar groups, exhibit structural diversity that significantly influences tea infusion color and taste. These compounds affect color through multiple mechanisms. For instance, cyanidin 6,7-diglucoside demonstrates a positive correlation with the L* value (r = 0.21). Its highest concentration in ML samples corresponds with their significantly elevated L* values compared to other samples. This may be attributed to the sugar groups of cyanidin 6,7-diglucoside, which disperse colloidal particles and prevent aggregation in aqueous solutions [41], thereby enhancing lightness. Quercetin 3-O-glucuronide, identified as a major ultraviolet-absorbing substance in plants [42], exhibits a negative correlation with the b* value (r = −0.2), suggesting that it may reduce yellowness through UV light absorption. Regarding taste, 6′-(4-hydroxycinnamoyl)astragaloside IV 4′-glucoside demonstrates a strong positive correlation with astringency (r = 0.85). This correlation may be attributed to the cinnamoyl group’s ability to enhance hydrophobic interactions [43], thereby intensifying the astringent perception. The CF sample contains the highest level of this compound, which likely contributes to its elevated astringency score. Rhodioloside 6,7-diglucoside exhibits a significant positive correlation with sweetness (r = 0.94). Its concentration is notably higher in the LJ sample compared to other samples. This compound may enhance sweetness perception by binding to sweet taste receptors (T1R2/T1R3) through hydrogen bonds formed by its disaccharide group [44], potentially explaining the LJ sample’s higher sweetness score.

##### Hydrolyzable Tannins

Hydrolyzable tannins are polyphenolic compounds formed via ester bonds connecting gallic acid or its derivatives to sugar groups. Concerning tea infusion color, 1,2,4,6-tetragalloyl glucose demonstrates a significant negative correlation with the L* value (r = −0.33), potentially due to its formation of large-particle colloids through hydrophobic aggregation, which reduces light scattering efficiency [45]. The ML sample exhibits significantly higher levels of 1,2,4,6-tetragalloyl glucose compared to other samples, which may contribute to its lowest L* value.

### 3.2. The Impact of Tea Cultivars on the Aroma of Black Tea

#### 3.2.1. Comparison of the Types and Compositions of Volatile Substances

Six tea samples were analyzed using GC-MS, resulting in the identification of 91 volatile compounds. These were classified into the following seven major categories: 13 alcohols, 21 acids/esters, 21 aldehydes, 10 ketones, 11 alkenes, 14 hydrocarbons, and 1 other compound (Table 3). Alcohols and aldehydes, which contribute significantly to black tea’s fresh, floral, fruity, and honey-like aromas [46,47] constituted the largest proportions across all samples, ranging from 18.4% to 33.69% and 22.83% to 27.58%, respectively, together accounting for over half of the total volatile composition. Esters (11.27–29.40%), ketones (5.18–19.90%), hydrocarbons (1.75–4.31%), and other compounds (0.34–1.17%) followed in descending order of abundance.

#### 3.2.2. Analysis of the Discrepancies in Aroma Substances

**Figure 6:**
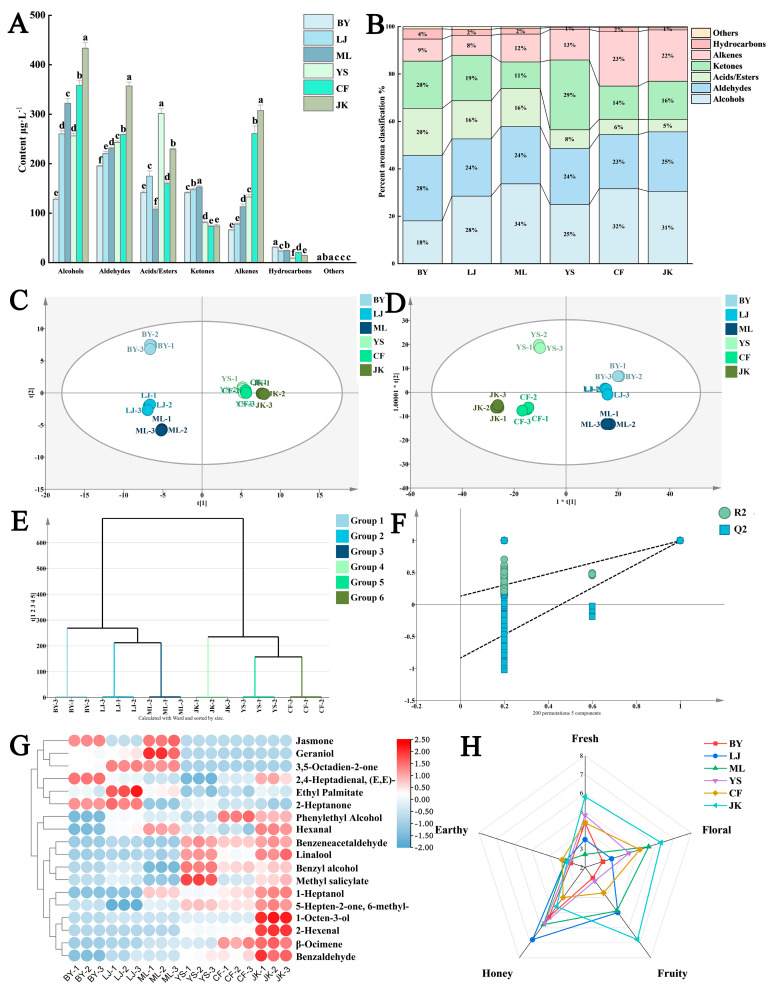
Types of volatile metabolites of black tea from different tea cultivars, multivariate statistical analysis, and key aroma differential substances: (**A**) Contents of volatile compounds in different categories; (**B**) Stacked column bar chart of aroma component percentages; (**C**) PCA analysis of aroma; (**D**) OPLS-DA analysis of aroma; (**E**) HCA analysis of aroma; (**F**) Permutation test; (**G**) Heat map of key differential aroma substances; (**H**) QDA radar chart of aroma. Note: Different letters indicate a statistically significant (*p* < 0.05) difference between groups.

Principal component analysis (PCA) was performed to examine differences in volatile substances among black teas from different tree cultivars. As shown in Figure 6C, samples from the same tea tree variety clustered together. BY, LJ, and ML samples were primarily located in the second and third quadrants, while YS, CF, and JK samples were distributed in the first and fourth quadrants. The six tea tree cultivars were generally divided into two distinct groups based on their volatile composition. This distribution pattern indicates significant differences in aroma substance types and concentrations among black teas from different tree cultivars. Furthermore, samples within each group demonstrated relatively high similarity in their aroma composition.

The results of hierarchical cluster analysis (HCA) (Figure 6E) reveal that aroma substances from different tea cultivars fall into two distinct categories. BY, LJ, and ML cluster in one category, with LJ and ML showing closer similarity in aroma profiles. JK, YS, and CF form the second category, where YS and CF demonstrate greater similarity in their aroma composition.

To enhance the separation and differentiation among samples from six tea cultivars, an orthogonal partial least squares discriminant analysis (OPLS-DA) model was applied to volatile compounds identified by GC-MS (Figure 6D). Results demonstrated excellent model performance with a cumulative explanatory variance of R^2^X = 0.994, R^2^Y = 0.997, and a predictive capacity of Q^2^ = 0.994, indicating high explanatory power and robust predictive performance.

To further investigate the differential volatile substances in black tea produced from different tea cultivars, an OPLS-DA model was applied to the GC-MS-analyzed volatile compounds. Based on VIP values from the model, 18 volatile substances with VIP > 1 were identified. These differential aroma compounds were visualized through a heat map. Results showed that (E,E)-2,4-heptadienal was prevalent in BY; (3E,5E)-octa-3,5-dien-2-one, ethyl palmitate, and 2-heptanone in LJ; jasmone, geraniol, and hexanal in ML; benzyl alcohol and methyl salicylate in YS; phenethyl alcohol in CF; and 1-octen-3-ol, β-ocimene, and benzaldehyde in JK.

#### 3.2.3. Screening and Analysis of Key Aroma-Contributing Substances

The contribution of aroma components to the overall volatile profile primarily depends on the following two critical factors: aroma threshold and concentration. The aroma threshold represents the minimum concentration at which a specific aroma compound can be detected by humans and is an indicator of its sensory relevance. The relative aroma activity value (OAV) serves as a key metric for evaluating the contribution of individual aroma compounds to the overall aroma profile. It is calculated as the ratio of the compound’s concentration to its corresponding aroma threshold in the medium [48]. By determining the OAV values of volatile substances in black teas produced from different tea cultivars, the relative contribution of each aroma component to the overall sensory perception can be quantitatively assessed. This approach enables the identification of key aroma components that significantly influence the sensory quality of black tea.

Based on the criteria of VIP > 1 and OAV ≥ 1, a total of 13 volatile aroma substances were identified (Table 4), the majority of which possess pleasant odors such as fresh floral and fruity scents. Phenylacetaldehyde, 1-octen-3-ol, β-ocimene, linalool, geraniol, and jasmone are recognized as contributors to the fresh and floral aromas in black tea. n-Heptanol, 2-hexenal, ethyl palmitate, and (3E,5E)-octa-3,5-dien-2-one constitute the fruity aroma components in tea. Methyl salicylate exhibits a minty and wintergreen odor, imparting a refreshing sensation to the tea infusion and also being beneficial for sweetness and floral aroma perception [49]. (E,E)-2,4-Heptadienal significantly contributes to the greenish flavor in black tea [50] and, at lower concentrations, enhances the fresh floral aroma [51]. Hexanal’s impact on tea aroma also depends on its concentration as follows: it imparts a greenish flavor at higher levels while providing a refreshing note at lower concentrations [47].

As depicted in Figure 6G, the BY sample exhibits a conspicuously higher concentration of (E,E)-2,4-heptadienal compared to other samples. This suggests that (E,E)-2,4-heptadienal may be the principal contributor to the slightly greenish odor characteristic of the BY sample. Furthermore, the remaining key aroma substances in the BY sample are present at relatively lower concentrations. This phenomenon might be attributed to the distinctive physiological traits of the albino tea plant variety, which results in a lower threshold and reduced quantities of terpene aroma precursor substances with floral and fruity notes [52]. The incomplete development of chloroplasts in albino tea plants disrupts normal terpenoid metabolism, limiting the transformation of these aroma precursors into significant black tea aroma compounds such as linalool during processing [53]. Research demonstrates that ethyl palmitate significantly influences the formation of fruity and sweet aromas in black tea [54]. This property likely explains the prominent sweet aroma characteristic of the LJ sample, where the high content of ethyl palmitate serves as the primary reason for this sensory attribute. Geraniol and jasmone are crucial aroma compounds in black tea, derived from the enzymatic hydrolysis of glycosides and lipid precursors, respectively [55,56]. These compounds exhibit rose and jasmine floral aroma characteristics, endowing black tea with a distinctive floral-dominated aroma profile. In the ML samples, elevated levels of geraniol and jasmone likely contribute to their pronounced floral characteristics. These compounds, derived from enzymatic hydrolysis of glycosides and lipid precursors, respectively, impart rose and jasmine floral notes to black tea. Methyl salicylate, which exhibits a mint-like refreshing quality, enhances sweetness and floral aroma when transformed from its precursor substances during processing. The relatively high content of methyl salicylate in YS samples may be a key factor in their distinct, refreshing aroma profile. Phenylacetaldehyde in black tea primarily originates from amino acid degradation and possesses a delightful narcissus-like floral scent. During black tea fermentation, amino acids are transformed into aldehyde compounds through both enzymatic reactions and non-enzymatic pathways, with phenylacetaldehyde being a significant product of this transformation [57]. In the YS, CF, and JK samples, phenylacetaldehyde content was significantly higher than in other samples, likely contributing to distinct aroma profile differences. Similarly, ocimene and linalool exhibited comparable patterns. Phenylacetaldehyde primarily originates from amino acid degradation during black tea fermentation, while β-ocimene and linalool are produced through enzymatic hydrolysis of glycoside precursors and terpene alcohol conversion, respectively. These compounds collectively impart black tea with its characteristic fresh floral aroma. Heptanol and 1-octene-3-ol are primarily formed through fatty acid degradation during black tea processing, particularly during fermentation when fatty acids undergo enzymatic oxidation to produce unsaturated alcohols. In the JK sample, these compounds are present at relatively high concentrations. Their presence contributes citrus-like fruity aromas and floral scents reminiscent of lavender and rose, potentially constituting a crucial source of the JK sample’s distinctive floral and fruity aroma profile.

High-quality black tea typically exhibits a complex floral and fruity aroma profile [58]. In this study, we observed that JK demonstrated superior comprehensive performance in terms of aroma quality, with significantly higher OAVs for four key aroma compounds compared to the other samples. Specifically, 1-Heptanol is a representative substance of the fruity aroma in black tea samples, with fresh, citrus-like aroma characteristics. Linalool, β-Ocimene, and 1-Octen-3-o are representative floral substances with high OAVs. In the Flaig et al. [51] study, aroma substances such as linalool were also found to be higher in the Jiukeng group cultivars. In addition, JK sample’s OAV of Benzeneacetaldehyde is also high among the six samples. As an important transformed aroma product, it has the characteristic smell of hyacinth flowers and daffodils and is a representative of special floral substances. Therefore, these five aromas were used as the research objects to explore their interactions with ORs, which provides a basis for this study of JK’s aroma quality analysis and aroma formation mechanism.

#### 3.2.4. Molecular Docking Analysis of the Combination and Interaction Between Aroma Substances and Aroma Receptors

##### The Binding Interaction Region Between ORs and Aroma Compounds

**Figure 7:**
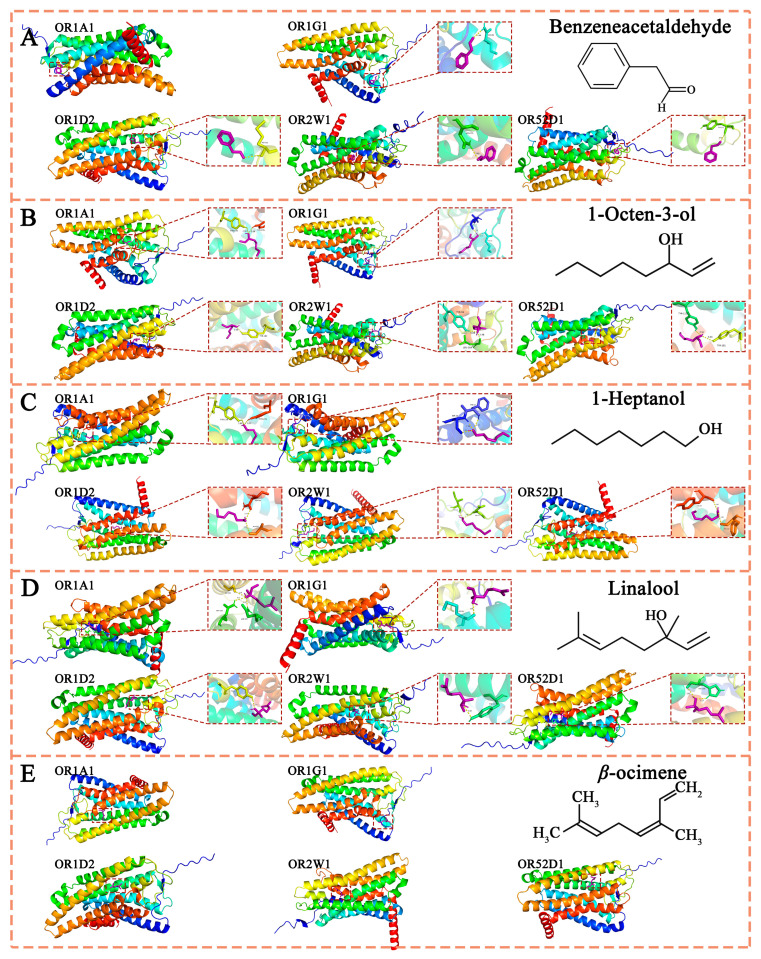
The molecular docking results of aroma substances and olfactory receptors: (**A**) 1-Octen-3-o; (**B**) 1-Heptanol; (**C**) Benzeneacetaldehyde; (**D**) β-Ocimene; and (**E**) linalool.

Among the approximately 390 human olfactory receptors (ORs), OR1A1, OR1D2, OR1G1, OR2W1, and OR52D1 are classified as broad-spectrum receptors capable of responding to diverse aroma compounds [59,60]. These receptors were selected as research subjects due to their ability to detect multiple odorants. Structurally, they share a common architecture featuring a seven-transmembrane (7-TM) domain, which forms the primary binding site for odorant molecules. The amino terminus (N-terminus) of these receptors is located on the extracellular side of the cell membrane, while the carboxyl terminus (C-terminus) faces the cytoplasmic side. The transmembrane helix is connected by three extracellular loops (EC1-EC3) and three intracellular loops (IC1-IC3) arranged alternately [61]. Each transmembrane segment of the five ORs (OR1A1, OR1G1, OR2W1, OR1D2, and OR52D1) contains between 19 and 24 amino acids, indicating moderate variability in sequence length and domain composition within this protein class. Comparative analysis of the amino acid sequences of these five olfactory receptors reveals both conserved regions and variable domains, reflecting structural diversity. This sequence diversity forms the structural basis for the binding specificity of olfactory receptors to various odorant molecules [62]. The third, fourth, and fifth transmembrane domains (TM3, TM4, and TM5) exhibit significant sequence variability and serve as key regions for olfactory receptor binding to aroma compounds. This diversity provides the structural basis for olfactory receptors to recognize multiple odor molecules, consistent with previous research [63,64]. Consequently, the TM3-TM5 regions are crucial binding sites for characteristic aroma substances. As shown in Figure 7, this study identified key residues for the binding of floral and fruity aroma substances in the JK sample, including Asn155 (TM4, OR1A1), Asn109 (TM3, OR1A1), Gly202 (TM5, OR1A1), Tyr104 (TM3, OR2W1), and Asn155 (TM3, OR2W1).

##### The Comparison of Binding Energies Between Different ORs and Aroma Substances

The binding energies between different olfactory receptors and aroma substances show significant variation, ranging from −5.2 kcal/mol to −3.25 kcal/mol (Table 5). These values indicate spontaneous binding between olfactory receptors and aroma compounds. Linalool demonstrates the strongest binding energy with OR1A1 and OR2W1 at −5.2 kcal/mol, suggesting high binding stability. Conversely, n-heptanol exhibits the weakest binding energy with OR2W1 at −3.25 kcal/mol, indicating lower binding stability. The binding affinity of the same aroma substance varies across different receptors. The average binding energies of 1-octen-3-ol, n-heptanol, phenylacetaldehyde, β-ocimene, and linalool with the five receptors are −3.98 kcal/mol, −3.49 kcal/mol, −4.53 kcal/mol, −4.93 kcal/mol, and −4.74 kcal/mol, respectively. These results suggest β-ocimene has the strongest receptor affinity, while n-heptanol has the weakest receptor affinity. From a molecular structural perspective, 1-octen-3-ol, phenylacetaldehyde, and linalool all contain C=C or C=O double-bond structures. These double bonds can interact with substituents (hydroxyl or amino groups) on the aromatic ring of receptor amino acid residues through p-π conjugation, thereby enhancing their interaction forces. Notably, 1-octen-3-ol exhibited binding energies of −4.69 kcal/mol with OR1G1 and −3.33 kcal/mol with OR1D2, representing a 40.8% difference—the largest observed binding energy difference. In contrast, β-ocimene demonstrated the smallest binding energy difference of 15.8%, with values of −5.13 kcal/mol with OR1G1 and −4.43 kcal/mol with OR52D1. The interaction regions and involved amino acid residues for the same aroma substance vary across the five receptors, contributing significantly to binding energy discrepancies. Higher binding energy indicates greater energy release during binding and stronger affinity between the aroma substance and the receptor. These affinity differences dictate the sequential activation of receptors by the aroma substance, ultimately determining its distinctive odor profile.

A single olfactory receptor can bind to diverse aroma substances with varying binding energies. For instance, OR1A1 exhibits binding energies of −4.29 kcal/mol with 1-octen−3-ol, −3.55 kcal/mol with n-heptanol, −4.52 kcal/mol with phenylacetaldehyde, −5.12 kcal/mol with β-ocimene, and −5.2 kcal/mol with linalool, representing a maximum difference ratio of 46.5%. Similarly, the maximum difference ratios for binding energies of different aroma substances with OR1G1, OR2W1, OR1D2, and OR52D1 are 26.4%, 60%, 46.5%, and 25.9%, respectively. These findings indicate that a single receptor possesses varying affinities when recognizing and binding to different aroma substances. Characteristic aroma molecules preferentially bind to receptors with stronger binding capacities, occupying specific surface areas. This mechanism enables different aroma molecules to exhibit distinct odor intensities and unique sensory profiles [65].

##### The Types of Interaction Forces Between Olfactory Receptors (ORs) and Aroma Substances

Table 6 lists key aroma substances and their corresponding olfactory receptor (OR) residues, including 1-octen-3-ol with Tyr178 and Tyr276 (OR1A1), n-heptanol with Phe17 and Ser18 (OR1G1), linalool with Tyr104 (OR2W1), and phenylacetaldehyde with Met181 (OR1D2). Most aroma substances form hydrogen bonds with ORs, a critical factor in aroma perception. Linalool, for example, forms hydrogen bonds with five receptors (OR1A1, OR1G1, OR2W1, OR1D2, and OR52D1) with binding energies ranging from −5.2 to −4.01 kcal/mol. The hydroxyl group in linalool interacts with amino acid residues such as Asn109/Asn155 (OR1A1), Tyr104 (OR2W1), Tyr182 (OR1D2), and His108 (OR52D1), forming hydrogen bonds with an average energy of −4.75 kcal/mol. These interactions demonstrate that hydrogen bonding significantly contributes to the binding affinity between aroma substances and their corresponding receptors.

The experimental data indicate a correlation between hydrogen bond quantity and binding capacity between aroma substances and olfactory receptors. For example, 1-octen-3-ol forms two hydrogen bonds with OR1A1, OR1G1, OR2W1, and OR52D1, with binding energies ranging from −4.29 to −3.44 kcal/mol. In contrast, it forms only one hydrogen bond with OR1D2, resulting in a lower binding energy of −3.33 kcal/mol. These findings suggest that hydrogen bonds significantly contribute to the affinity between aroma substances and receptors. Notably, β-ocimene exhibits no hydrogen bonding with the five receptors, likely due to the absence of interactive functional groups such as hydroxyl or carboxyl in its molecular structure. Despite this, β-ocimene demonstrates relatively strong binding affinity (average energy −4.93 kcal/mol), indicating that factors beyond hydrogen bonds influence binding stability between aroma substances and receptors. Although no hydrogen bonds are present in these complexes, hydrophobic interactions occur between β-ocimene and hydrophobic amino acid residues in OR1G1 (Leu14, Leu15, Gly16, Phe17, Ser18, Gln24, Leu27, Asn84, Ile85, Gln88, Ser89, and Gln90) and OR52D1 (Ala206, Leu207, Met210, Gly211, Tyr255, Ala258, Phe259, and Phe262). These residues form contiguous hydrophobic regions that stabilize the binding of β-ocimene, thereby maintaining its floral and neroli oil aroma characteristics. Phenylacetaldehyde also undergoes hydrophobic interactions with amino acid residues such as Leu14, Gly16, Phe17, Ser18, Gln24, and Leu27 in OR1G1 (Table 6), ensuring stable binding to the olfactory receptor. Research indicates that hydrophobic amino acid residues create a hydrophobic environment for aroma substances, thereby facilitating their stable binding. Amino acid residues with benzene ring structures—such as Trp, Tyr, and Phe—are particularly prone to hydrophobic interactions with aroma substances. For example, residues like Tyr178 and Phe177 in OR1A1 and Phe17 in OR1G1 enhance the interaction between linalool and phenylacetaldehyde with their respective olfactory receptors. This finding aligns with previous research, indicating that hydrophobic interactions between amino acid residues with benzene ring structures and aroma substances enhance the binding affinity of olfactory receptors for these compounds [66]. Consequently, hydrophobic interactions play a crucial role in stabilizing the receptor-aroma substance complex [66,67].

## 4. Conclusions

The results demonstrated that amino acids and their derivatives generally enhanced brightness (L*) and predominantly contributed to the perception of freshness and sweetness. Catechins and their derivatives primarily imparted a bitter taste to the tea broth, while phenolic acids, flavonoid glycosides, their derivatives, and hydrolysable tannins exhibited diverse effects on soup color and taste characteristics. Notably, the JK sample exhibited high levels of non-ester-type catechins, soluble sugars, theaflavin B, proline-glutamine dipeptide (Pro-Gln), and other components, which were strongly correlated with sweetness. This corresponded to the highest sweetness score in the sensory evaluation of the sample. Theaflavin C, a key substance regulating the reddish hue (a* value), was found in the highest concentration in the JK sample. The abundance of these metabolites significantly influenced the color and taste characteristics of the tea broth, resulting in the JK sample achieving a higher sensory score compared to the other samples. Additionally, the JK sample contained elevated levels of volatile compounds such as 1-octen-3-ol, 1-heptanol, phenylacetaldehyde, β-Ocimene, and linalool, which conferred a distinctive fresh citrus and floral aroma, setting it apart from the other samples. Molecular docking technology was employed to explore the interaction mechanisms between these characteristic aroma compounds and five broad-spectrum olfactory receptors, offering novel insights into the formation of the floral and fruity aroma and flavor of the JK sample. In this study, the sensory quality and chemical composition of black tea derived from six tea plant cultivars (Baiye 1#, Longjing 43#, Maolv, Yingshuang, Cuifeng, and Jiukeng group cultivars) grown in the Jingshan area were systematically analyzed. It was determined that the Jiukeng group cultivars (JK sample) excelled in terms of soup reddish hue, sweetness, floral and fruity aroma, and overall sensory score. These findings provide valuable guidance for selecting tea cultivars suitable for black tea production in the Jingshan area.

## Figures and Tables

**Table 1:** The gradient concentrations and scores of taste standard compounds.

Taste	Standards (g/L)	Scores					
		0	1	2	3	4	5
Umami	Monosodium glutamate	0	0.16	0.32	0.48	0.64	0.8
Sweetness	Sucrose	0	2	4	6	8	10
Astringency	EGCG	0	0.2	0.4	0.6	0.8	1
Bitterness	Caffeine	0	0.3	0.6	0.9	1.2	1.5

**Table 2:** The key color and taste-related compounds contributing to the color and taste of different tea tree cultivar samples.

ID	Compounds	m/z	VIP (LAB)	VIP (Taste)
pos_870	Pro-Gln	226.12	1.34	<1
pos_1524	Histidylleucine	268.15	2.38	2.16
pos_1716	2,6-Digalloylglucose	502.12	<1	1.26
pos_1813	Delphinidin 3-caffeoylglucoside	666.09	<1	1.05
pos_2297	1,6-Digalloyl-β-D-glucopyranose	467.08	<1	1.38
pos_2892	Kaempferol 3-o-rhamninoside	741.22	1.05	1.11
pos_3107	Indole-3-acetyl-L-aspartic acid	291.1	1.27	1.4
pos_3142	Kaempferol 7-(6″-galloylglucoside)	601.12	2.4	1.81
pos_3914	Delphinidin 3-(6-p-coumaroylgalactoside)	611.14	2.18	1.54
pos_4371	Glu-Leu-His-Glu	527.25	1.31	<1
pos_5630	Propyl gallate	177.05	1.08	1.1
pos_7437	Gly-Ala-Pro-Met-Phe	620.32	1.31	1.01
pos_8384	Gly-Ser-Pro-Met-Phe	636.32	1.68	1.06
pos_9659	Artemetin	389.12	1.34	1.33
pos_9964	Neotheaflavin 3-gallate	734.17	1.59	1.92
pos_9969	Biflorin	359.08	1.05	1.23
pos_10356	Schinifoline	283.14	2.14	2.88
pos_10419	(-)-Epigallocatechin 3-cinnamate	437.12	<1	1.01
pos_11115	Ile-His-Ala-Glu	469.24	1.62	1.21
pos_11116	Glu-Phe-Arg	451.23	1.57	1.22
pos_11262	His-His-His	412.19	1.03	1.47
pos_12140	2′-C-Methylmyricetin 3-rhamnoside 5′-gallate	595.11	3.12	3.99
pos_12770	Quercetin 3-arabinoside 7-glucoside	601.12	1.89	1.82
pos_12874	(-)-Epigallocatechin 3,4′-di-gallate	611.11	1.45	1.63
pos_12943	Kaempferol 3-α-L-arabinofuranoside	441.08	<1	1.08
pos_13329	Pretyrosine	192.07	<1	1.04
pos_13370	1,2,6-Trigalloylglucose	654.13	<1	1.37
pos_13602	Val-Trp	321.19	<1	1.02
pos_13657	Trans-p-Coumaroyl β-D-glucopyranoside	344.13	<1	1.35
pos_13662	Phe-Glu	295.13	1.01	1.09
pos_13667	Leu-Hyp	227.14	1.14	1.22
pos_14125	Ile-Thr-Trp	401.22	1.07	<1
pos_14959	Asn-Pro	271.14	2.52	1.26
pos_15431	Citrulline	158.09	1.31	<1
pos_15692	Peonidin 3-rutinoside	627.22	1.1	<1
neg_1790	Phe-Thr	265.12	<1	1.04
neg_2521	Feruloylquinic Acid	367.1	1.22	<1
neg_2957	Quercetin 3-O-xylosyl-glucuronide	631.1	3.3	2.64
neg_2985	1,2,4-Trigalloyl-β-D-glucopyranose	635.09	<1	1.29
neg_3696	Vitexin	431.1	<1	1.07
neg_3758	Myricitrin	927.19	1.08	1.36
neg_3960	4-Hydroxybenzoyl glucose	281.07	1.06	<1
neg_3990	Gly-Phe	203.08	<1	1.16
neg_3991	N-acetyl-l-tryptophan	245.09	<1	1.12
neg_4461	3-(4-Hydroxybenzoyl)epicatechin	455.1	1.15	1.07
neg_4541	Catechin 7-glucoside	497.13	1.05	1.03
neg_4626	Trans-3-Hydroxycinnamate	163.04	<1	1.07
neg_4999	Prunin 6″-O-gallate	585.13	1.96	1.49
neg_5195	Hexyl glucoside	245.14	1.3	1.05
neg_5379	Quercetin 3-(3-p-coumaroylglucoside)	609.13	1.42	<1
neg_5542	Theaflavate B	699.14	1.32	1.52
neg_5653	Caryatin glucoside	551.14	1.19	1.36
neg_5737	(1S,2S,4R,5S,7S)-2,5,7-Fenchanetriol 2-O-b-D-glucoside	347.17	1.27	1.17
neg_5859	Luteolin 7-O-(2-apiosyl-4-glucosyl-6-malonyl)-glucoside	849.17	1.77	1.94
neg_6221	Ligustroside	569.19	2.27	1.36
neg_6234	Ononin	429.12	1.66	2.27
neg_6659	Formononetin 7-(6″-methylmalonylglucoside)	551.12	2.28	1.83
neg_9096	Lys-Trp-Lys	459.28	1.13	<1
neg_9708	Canavalioside	605.28	<1	1.19
neg_11484	Arg-Val-Lys	400.27	1.94	1.41
neg_12563	6-Acetyl-D-glucose	221.07	1.28	<1
neg_13036	3′,4,4′-Trihydroxypulvinone	311.06	1.85	1.76
neg_13240	7-Hydroxybutylidenephthalide 7-(6-malonylglucoside)	451.13	1.33	<1
neg_13437	Lys-Asp-Glu-Leu	524.24	<1	1.04
neg_13708	Taxifolin 3-α-L-arabinofuranoside	435.09	<1	1.36
neg_14965	Quercetin 3-O-glucuronide	477.07	1.63	2.69
neg_15158	3,5-Digalloylepicatechin	593.09	1.09	1.36
neg_16109	6′’-(4-Hydroxycinnamoyl)astragalin 4′-glucoside	737.17	1.02	1.68
neg_16123	(S)-Mandelic acid O-β-D-Glucopyranoside	295.08	<1	1.33
neg_16316	Epigallocatechin 3,5-digallate	609.09	1.2	1.16
neg_16336	1,2,4,6-tetragalloylglucose	787.1	1.15	1.36
neg_16411	(-)-Epigallocatechin 3-(4-methyl-gallate)	471.09	1.21	1.03
neg_16511	Quercetin 3-(2-galloylglucoside)	615.1	1.79	1.2
neg_16755	Feruloyl quinic acid	367.1	1.08	<1
neg_17083	Carthamidin 6,7-diglucoside	611.16	<1	1.01
neg_18318	Asp-Val	231.1	1.22	<1
pos_11536	Theaflavin-3-gallate	717.15	1.39	1.33
neg_1932	Theacitrin A	759.12	1.61	1.29
neg_2903	Theacitrin C	911.13	1.68	1.72
pos_12345	Theanine	174.2	2.47	3.36
pos_12346	EGC	457.08	3.41	3.11
pos_12347	GC	305.07	2.21	2.9
pos_12348	C	289.06	3.6	3.63
pos_12349	GCG	487.08	2.56	1.95

**Table 3:** Key aroma substances of black tea of different tea cultivar.

No.	CAS		RI	BY	LJ	ML	YS	CF	JK
	Alcoholic compounds								
1	1576-87-0	trans-2-Pentenal	1228	4.53 ± 0.25 ^c^	9.06 ± 0.36 ^a^	4.62 ± 0.48 ^c^	2.83 ± 0.31 ^d^	6.75 ± 0.27 ^b^	8.55 ± 0.91 ^a^
2	10340-23-5	cis-3-Nonen-1-ol	1565	0.87 ± 0.1 ^a^	0.79 ± 0.09 ^a^	0.57 ± 0.09 ^b^	0	0	0
3	106-25-2	Nerol	1255	0	0	0	2.21 ± 0.17 ^b^	1.53 ± 0.05 ^c^	4.61 ± 0.34 ^a^
4	96-76-4	2,4-Di-tert-butylphenol	1518	0.59 ± 0.02 ^a^	0.46 ± 0.07 ^b^	0	0.45 ± 0.06 ^b^	0	0
5	40716-66-3	1,6,10-Dodecatrien-3-ol, 3,7,11-trimethyl-, (E)-	1175	0.61 ± 0.02 ^b^	2.39 ± 0.16 ^a^	0	0	0	0
6	77-53-2	Cedrol	1600	1.15 ± 0.14 ^a^	0.24 ± 0.03 ^b^	0	0	0	0
7	481-34-5	α-Cadinol	1190	0.52 ± 0.02 ^b^	0	0	0	0.61 ± 0.04 ^a^	0
8	111-70-6	1-Heptanol	973	0	0	43.94 ± 0.77 ^b^	31.13 ± 0.93 ^d^	39.96 ± 2.65 ^c^	57.29 ± 1.94 ^a^
9	3391-86-4	1-Octen-3-ol	982	0	7.07 ± 0.11 ^c^	5.61 ± 0.22 ^c^	12.64 ± 0.1 ^b^	11.46 ± 0.32 ^b^	73.38 ± 2.26 ^a^
10	100-51-6	Benzyl alcohol	1037	29.76 ± 0.43 ^d^	40.44 ± 0.48 ^c^	12.75 ± 0.51 ^e^	80.5 ± 1.03 ^a^	62.52 ± 2.36 ^b^	62.68 ± 3.93 ^b^
11	78-70-6	Linalool	1102	28.17 ± 1.07 ^c^	30.11 ± 1.16 ^c^	30.96 ± 3.37 ^c^	69.32 ± 0.75 ^a^	50.72 ± 1.83 ^b^	72.86 ± 3.46 ^a^
12	60-12-8	Phenylethyl alcohol	1116	0	99.26 ± 1.77 ^c^	76.94 ± 5.46 ^d^	43.3 ± 3.16 ^e^	176.01 ± 6.33 ^a^	142.75 ± 2.36 ^b^
13	106-24-1	Geraniol	1256	61.37 ± 3.33 ^b^	70.05 ± 5.61 ^b^	146.44 ± 14.06 ^a^	13.36 ± 0.94 ^c^	8.55 ± 1.43 ^c^	11.18 ± 0.37 ^c^
	Aldehydic compounds								
14	111-71-7	Heptanal	903	2.46 ± 0.25 ^b^	1.22 ± 0.08 ^d^	3.21 ± 0.27 ^a^	0	1.49 ± 0.15 ^d^	1.97 ± 0.1 ^c^
15	824-22-6	4-methylindan	803	0	0	3.99 ± 0.24 ^b^	3.77 ± 0.23 ^b^	3.99 ± 0.21 ^b^	4.66 ± 0.26 ^a^
16	55722-59-3	3,6-Octadienal, 3,7-dimethyl-	855	0	0	2.44 ± 0.25 ^d^	3.71 ± 0.26 ^c^	5.6 ± 0.11 ^a^	4.46 ± 0.42 ^b^
17	116-26-7	Safranal	930	0.98 ± 0.11 ^c^	1.02 ± 0.07 ^c^	2.06 ± 0.13 ^a^	0.44 ± 0.04 ^d^	0.56 ± 0.03 ^d^	1.35 ± 0.08 ^b^
18	112-31-2	Decyl aldehyde	1206	0.52 ± 0.03 ^c^	0	0	1.1 ± 0.14 ^b^	1.34 ± 0.15 ^a^	1.13 ± 0.09 ^b^
19	15764-16-6	2,4-Dimethylbenzaldehyde	1150	0.52 ± 0.03 ^c^	0	0	1.1 ± 0.14 ^b^	1.34 ± 0.15 ^a^	1.13 ± 0.09 ^b^
20	5910-87-2	2,4-Nonadienal, (E,E)-	1214	0.83 ± 0.09 ^b^	0.93 ± 0.13 ^b^	1.3 ± 0.16 ^a^	0	0	0
21	432-25-7	Pentadeuterio--cyclocitral	1260	0	0.67 ± 0.08 ^c^	1 ± 0.07 ^b^	2.25 ± 0.21 ^a^	0.67 ± 0.07 ^c^	0
22	106-26-3	Neral	1290	0	0	0	1.19 ± 0.13 ^c^	2.36 ± 0.13 ^b^	3 ± 0.15 ^a^
23	26643-91-4	4-methyl-2-phenyl-1-pentenal	1315	0	1.96 ± 0.17 ^a^	1.94 ± 0.18 ^a^	1.29 ± 0.03 ^c^	1.57 ± 0.14 ^b^	1.74 ± 0.12 ^ab^
24	13019-16-4	(E)-2-butyloct-2-enal	1040	0.98 ± 0.09 ^d^	1.69 ± 0.12 ^c^	2.88 ± 0.26 ^b^	3.34 ± 0.27 ^a^	3.27 ± 0.2 ^a^	2 ± 0.2 ^c^
25	21834-92-4	Cocal	960	7.84 ± 0.75 ^a^	1.12 ± 0.03 ^c^	0.87 ± 0.13 ^c^	0	0	2.31 ± 0.42 ^b^
26	6728-26-3	2-Hexenal, (E)-	960	7.05 ± 0.15 ^a^	7.02 ± 0.62 ^a^	6.95 ± 0.25 ^a^	6.13 ± 0.4 ^b^	5.21 ± 0.1 ^c^	7.18 ± 0.33 ^a^
27	4313-03-5	2,4-Heptadienal, (E,E)-	1012	56.73 ± 0.35 ^a^	36.21 ± 1.24 ^c^	28.1 ± 1.02 ^e^	13.28 ± 0.98 ^f^	31.73 ± 0.42 ^d^	47.73 ± 3.58 ^b^
28	122-78-1	Benzeneacetaldehyde	1044	0	3.66 ± 0.21 ^c^	0	25.71 ± 1.49 ^a^	21.11 ± 0.05 ^b^	25.58 ± 1.25 ^a^
29	141-27-5	2,6-Octadienal, 3,7-dimethyl-, (E)-	1271	0	0	4.67 ± 0.22 ^c^	10.02 ± 0.74 ^b^	12.74 ± 1.37 ^a^	12.54 ± 0.48 ^a^
30	5392-40-5	Citral	1240	2.92 ± 0.16 ^e^	3.02 ± 0.26 ^e^	5.22 ± 0.1 ^d^	8.22 ± 0.29 ^b^	7.35 ± 0.14 ^c^	15.75 ± 0.48 ^a^
31	66-25-1	Hexanal	803	30.8 ± 0.94 ^e^	51.32 ± 0.9 ^b^	61.51 ± 1.07 ^a^	44.69 ± 2.28 ^c^	39.09 ± 4.35 ^d^	64.09 ± 1 ^a^
32	505-57-7	2-Hexenal	855	0	0	0	7.82 ± 0.49 ^c^	8.65 ± 0.48 ^b^	22.92 ± 0.55 ^a^
33	100-52-7	Benzaldehyde	960	69.34 ± 1.31 ^d^	86.06 ± 4.94 ^c^	89.08 ± 0.61 ^c^	104.93 ± 2.9 ^b^	108.54 ± 3.67 ^b^	135.77 ± 5.8 ^a^
34	124-13-0	Octanal	1003	9.68 ± 0.88 ^b^	14.89 ± 1.03 ^a^	10.45 ± 0.56 ^b^	0	0	0
	Acid esters								
35	106-70-7	Hexanoic acid, methyl ester	928	3.63 ± 0.23 ^c^	4.1 ± 0.19 ^c^	3.71 ± 0.51 ^c^	5.84 ± 0.69 ^b^	6.02 ± 0.74 ^b^	9.67 ± 0.48 ^a^
36	16491-36-4	3-hexenylbutyrate	1187	2.78 ± 0.18 ^a^	2.22 ± 0.13 ^b^	2.12 ± 0.15 ^b^	2.97 ± 0.21 ^a^	2.67 ± 0.12 ^a^	2.07 ± 0.19 ^b^
37	35154-45-1	cis-3-Hexenyl isovalerate	1150	4.28 ± 0.25 ^a^	2.89 ± 0.15 ^c^	3.36 ± 0.3 ^b^	2.57 ± 0.32 ^cd^	2.43 ± 0.15 ^d^	3.55 ± 0.21 ^b^
38	1189-09-9	E-Methylgeranate	1380	0	1.29 ± 0.11 ^a^	0.79 ± 0.07 ^b^	0	1.28 ± 0.1 ^a^	0
39	104-61-0	Nonanolactone	1363	1.7 ± 0.12 ^a^	1.33 ± 0.07 ^b^	1.29 ± 0.07 ^b^	1.36 ± 0.12 ^b^	0	0
40	31501-11-8	cis-3-Hexenyl hexanoate	1120	0.53 ± 0.06 ^b^	1 ± 0.11 ^a^	0.91 ± 0.1 ^a^	0	0	0
41	6378-65-0	Hexyl hexanoate	1194	0.66 ± 0.08 ^d^	0	1.42 ± 0.11 ^c^	1.57 ± 0.03 ^b^	1.79 ± 0.09 ^a^	1.65 ± 0.1 ^ab^
42	53398-86-0	trans-2-Hexenyl Hexanoate	1250	0.24 ± 0.08 ^c^	0.42 ± 0.07 ^a^	0.34 ± 0.01 ^b^	0.24 ± 0.01 ^c^	0.24 ± 0.04 ^c^	0
43	103-52-6	Phenylethyl butyrate	1170	0.82 ± 0.06 ^d^	1.19 ± 0.13 ^a^	1.14 ± 0.1 ^ab^	0.9 ± 0.08 ^cd^	1.02 ± 0.06 ^bc^	1.15 ± 0.07 ^ab^
44	17092-92-1	Dihydroactinidiolide	1360	0.42 ± 0.04 ^c^	2.18 ± 0.11 ^b^	2.78 ± 0.07 ^a^	0	0	0
45	84-66-2	Diethyl Phthalate	1560	2.45 ± 0.06 ^a^	0	1.06 ± 0.06 ^c^	0.54 ± 0.05 ^d^	1.48 ± 0.02 ^b^	0.59 ± 0.05 ^d^
46	1211-29-6	Methyl jasmonate	1681	0	0	0.87 ± 0.02 ^b^	0.39 ± 0.01 ^d^	1.21 ± 0.08 ^a^	0.65 ± 0.1 ^c^
47	124-06-1	Ethyl myristate	1599	0.36 ± 0.03 ^b^	0.11 ± 0.01 ^e^	0.25 ± 0.01 ^c^	0.19 ± 0.01 ^d^	0.22 ± 0.02 ^c^	0.4 ± 0.01 ^a^
48	112-39-0	Hexadecanoic acid, methyl ester	1796	0	0	0	0.24 ± 0.02 ^a^	0.11 ± 0.02 ^b^	0.05 ± 0.01 ^c^
49	84-74-2	Dibutyl phthalate	2000+	0.49 ± 0.09 ^b^	0.58 ± 0.05 ^a^	0.55 ± 0.05 ^ab^	0	0	0
50	111-61-5	Ethyl stearate	1994	0	0	0	3.9 ± 0.53 ^b^	5.6 ± 0.76 ^a^	5.75 ± 0.89 ^a^
51	628-97-7	Ethyl Palmitate	2193	32.47 ± 1.48 ^c^	43.94 ± 1.66 ^a^	34.95 ± 0.94 ^b^	26.79 ± 1.54 ^d^	28.85 ± 1.22 ^d^	31.92 ± 1.1 ^c^
52	119-36-8	Methyl salicylate	1194	88.31 ± 2.43 ^d^	115.04 ± 12.11 ^c^	53 ± 3.87 ^e^	252.19 ± 7.88 ^a^	104.62 ± 3.29 ^c^	170.28 ± 2.63 ^b^
53	112-05-0	Nonanoic acid	1311	0	0	0	0.97 ± 0.15 ^a^	0.57 ± 0.04 ^c^	0.83 ± 0.09 ^b^
54	459-80-3	Geranic acid	1170	0	0.22 ± 0.03 ^c^	0	0.5 ± 0.02 ^a^	0.34 ± 0.01 ^b^	0.22 ± 0.03 ^c^
55	57-10-3	Palmitic acid	1994	1.15 ± 0.1 ^b^	0	1.91 ± 0.08 ^a^	0	0.23 ± 0.02 ^c^	0
	Hydrocarbons								
56	17301-23-4	Undecane, 2,6-dimethyl-	1332	4.21 ± 0.23 ^bc^	4.4 ± 0.25 ^b^	2.86 ± 0.15 ^d^	3.84 ± 0.21 ^c^	4.12 ± 0.22 ^bc^	4.91 ± 0.46 ^a^
57	91-57-6	2-Methylnaphthalene	1315	0.63 ± 0.07 ^b^	0.81 ± 0.06 ^a^	0.84 ± 0.13 ^a^	0	0	0.9 ± 0.08 ^a^
58	35599-77-0	1-Iodotridecane	1400	2.15 ± 0.15 ^b^	4.19 ± 0.13 ^a^	1.26 ± 0.07 ^c^	0	0.29 ± 0.02 ^e^	0.91 ± 0.16 ^d^
59	294-62-2	Cyclododecane	1420	3.47 ± 0.1 ^b^	3.5 ± 0.2 ^b^	2.72 ± 0.08 ^d^	3.09 ± 0.06 ^c^	2.88 ± 0.03 ^cd^	4.05 ± 0.25 ^a^
60	544-77-4	Hexadecyl iodide	1400	4.73 ± 0.19 ^b^	0	0.18 ± 0.02 ^d^	0	6.03 ± 0.13 ^a^	0.47 ± 0.05 ^c^
61	629-99-2	Pentacosane	1600	2.66 ± 0.11 ^a^	0.71 ± 0.08 ^c^	0	0.75 ± 0.07 ^c^	1.63 ± 0.15 ^b^	0.69 ± 0.08 ^c^
62	1560-92-5	Hexadecane, 2-methyl-	1800	0.45 ± 0.03 ^c^	2.18 ± 0.11 ^b^	2.78 ± 0.07 ^a^	0	0	0
63	27458-90-8	Tert-Dodecyl Disulfide	2000	1.11 ± 0.11 ^bc^	1.22 ± 0.1 ^b^	2.72 ± 0.18 ^a^	0	0.99 ± 0.14 ^c^	0
64	18435-45-5	1-Nonadecene	2200	1.86 ± 0.05 ^a^	1.51 ± 0.04 ^b^	0	0	1.55 ± 0.1 ^b^	0
65	544-76-3	Hexadecane	1200	0.74 ± 0.06 ^c^	3.5 ± 0.09 ^a^	0.97 ± 0.07 ^b^	0.41 ± 0.06 ^d^	0.68 ± 0.04 ^c^	0.23 ± 0.03 ^e^
66	7390-81-0	1,2-Epoxyoctadecane	1500	0	0	0	0	0.94 ± 0.06 ^a^	0.62 ± 0.06 ^b^
67	3075-84-1	2,2′,5,4′-Tetramethylbiphenyl	1700	0.82 ± 0.1 ^b^	0.27 ± 0.02 ^c^	1.55 ± 0.1 ^a^	0.26 ± 0.02 ^c^	0.28 ± 0.02 ^c^	0.21 ± 0.02 ^c^
68	629-94-7	Heneicosane	1900	6.6 ± 0.29 ^a^	0	6.42 ± 0.25 ^a^	0	0	0
69	112-88-9	1-Octadecene	1100	0	0.19 ± 0.02 ^c^	0.44 ± 0.03 ^b^	0	0.46 ± 0.02 ^b^	0.78 ± 0.13 ^a^
	Alkenic compounds								
70	5989-27-5	D-Limonene	1028	3.63 ± 0.23 ^c^	4.1 ± 0.19 ^c^	3.71 ± 0.51 ^c^	5.84 ± 0.69 ^b^	6.02 ± 0.74 ^b^	9.67 ± 0.48 ^a^
71	535-77-3	M-cymene	1024	2.01 ± 0.12 ^b^	4 ± 0.13 ^a^	2 ± 0.08 ^b^	1.98 ± 0.11 ^b^	0	0
72	7216-56-0	2,4,6-Octatriene, 2,6-dimethyl-, (E,Z)-	1415	1.47 ± 0.09 ^c^	1.19 ± 0.11 ^d^	0.79 ± 0.06 ^e^	4.6 ± 0.29 ^a^	0	1.97 ± 0.16 ^b^
73	99-86-5	α-Terpinene	1016	5.98 ± 0.33 ^a^	3.05 ± 0.18 ^c^	2.14 ± 0.14 ^d^	1.73 ± 0.12 ^d^	2.81 ± 0.43 ^c^	4.81 ± 0.31 ^b^
74	17699-14-8	(-)-α-cubebene	1349	2.33 ± 0.18 ^a^	0	0	0	0.38 ± 0.04 ^c^	1.57 ± 0.07 ^b^
75	475-20-7	Longifolene	1380	0	0	1.59 ± 0.08 ^a^	0.17 ± 0.02 ^c^	0.28 ± 0.06 ^c^	0.69 ± 0.13 ^b^
76	469-61-4	(-)-α-cedrene	1410	0.7 ± 0.15 ^d^	0.89 ± 0.1 ^cd^	0	2.72 ± 0.11 ^a^	0.96 ± 0.11 ^c^	1.42 ± 0.11 ^b^
77	21391-99-1	α-calacorene	1405	0.29 ± 0.01 ^e^	0.81 ± 0.06 ^c^	0.54 ± 0.05 ^d^	1.22 ± 0.06 ^a^	1.08 ± 0.07 ^b^	0.12 ± 0.02 ^f^
78	3856-25-5	Copaene	1420	0	0	0	4.32 ± 0.14 ^c^	10.03 ± 0.83 ^b^	13.13 ± 0.39 ^a^
79	123-35-3	Myrcene	991	0	0	0	5.89 ± 0.19 ^b^	5.43 ± 0.16 ^b^	13.64 ± 0.73 ^a^
80	13877-91-3	.beta.-Ocimene	1036	50.86 ± 0.38 ^d^	66.04 ± 2.13 ^d^	102.92 ± 5.76 ^c^	108.22 ± 5.41 ^c^	237.53 ± 15.83 ^b^	266.83 ± 11.41 ^a^
	Ketones compounds								
81	14309-57-0	3-Nonen-2-one	1013	1.02 ± 0.18 ^c^	1.68 ± 0.13 ^a^	1.36 ± 0.1 ^b^	1 ± 0.17 ^c^	0.89 ± 0.1 ^c^	1.68 ± 0.17 ^a^
82	127-41-3	α-Ionone	1422	0	0	1.08 ± 0.06 ^b^	1.76 ± 0.16 ^a^	0	0
83	6901-97-9	1-cyclohexene	1480	0	0	0.19 ± 0.01 ^d^	0.67 ± 0.03 ^c^	0.83 ± 0.05 ^b^	1.04 ± 0.06 ^a^
84	689-67-8	Geranylacetone	1453	0.33 ± 0.03 ^b^	0.59 ± 0.06 ^a^	0.59 ± 0.03 ^a^	0	0	0
85	79-77-6	β-Lonone	1480	0	0.59 ± 0.05 ^d^	3.51 ± 0.07 ^b^	0.82 ± 0.09 ^c^	4.06 ± 0.22 ^a^	0.77 ± 0.09 ^cd^
86	127-41-3	α-Ionone	1422	1.55 ± 0.05 ^a^	0	0	0.68 ± 0.1 ^b^	0.82 ± 0.12 ^b^	0.69 ± 0.13 ^b^
87	110-93-0	5-Hepten-2-one, 6-methyl-	887	15.64 ± 0.39 ^e^	0	20.19 ± 0.98 ^d^	32.57 ± 0.32 ^b^	29 ± 0.15 ^c^	38.45 ± 1.35 ^a^
88	488-10-8	Jasmone	1404	21.38 ± 0.44 ^b^	1.1 ± 0.12 ^c^	23.62 ± 1.04 ^a^	0	0	0
89	110-43-0	2-Heptanone	887	60.78 ± 0.22 ^b^	64.17 ± 1.6 ^a^	27.22 ± 0.38 ^f^	42.83 ± 2.23 ^c^	37.39 ± 1.05 ^d^	30.94 ± 2.87 ^e^
90	38284-27-4	3,5-Octadien-2-one	1041	40.02 ± 1.63 ^c^	78.81 ± 1.66 ^a^	74.61 ± 1.92 ^b^	0	0	0
	Others								
91	75-18-3	Dimethyl sulfide	<600	0	0	0	4.05 ± 0.19 ^b^	3.83 ± 0.28 ^b^	6.32 ± 0.45 ^a^

Note: Different letters indicate a statistically significant (*p* < 0.05) difference between groups.

**Table 4:** Odor activity values (OAVs) of key aroma compounds in black tea of different tea cultivars.

Compounds	Threshold	OAVS						Odor Quality
		BY	LJ	ML	YS	CF	JK	
1-Heptanol	0.76	0	0	57.81	40.96	52.58	75.38	Fresh, fatty, citrus-like
1-Octen-3-ol	1	0	7.07	5.61	12.64	11.46	73.38	Mushroom, lavender, rose
2,4-Heptadienal, (E,E)-	0.057	995.32	635.32	493.04	233.04	556.67	837.31	Fatty, green
Benzeneacetaldehyde	4	0	0.91	0	6.43	5.28	6.4	Floral, daffodil
β-Ocimene	2	25.43	33.02	51.46	54.11	118.76	133.42	Floral
Linalool	0.58	48.57	51.91	53.37	119.52	87.44	125.62	Floral
Geraniol	1.1	55.79	63.68	133.13	12.14	7.77	10.16	Floral
Jasmone	7	3.05	0.16	3.37	0	0	0	Woody, herbaceous, jasmine
Ethyl Palmitate	1	32.47	43.94	34.95	26.79	28.85	31.92	Waxy, fruity, creamy
Hexanal	2.4	12.83	21.38	25.63	18.62	16.29	26.7	grassy
2-Hexenal	0.03	0	0	0	260.78	288.33	763.89	Fruity, grassy
Methyl salicylate	40	2.21	2.88	1.33	6.3	2.62	4.42	Minty, wintergreen-like
3,5-Octadien-2-one	0.15	266.78	525.38	497.38	0	0	0	Fruity, fatty, mushroom

**Table 5:** Summary of the binding energy between olfactory receptors and ligands.

Receptor		Building Energy (kcal/mol)				
	Ligand	OR1A1	OR1G1	OR2W1	OR1D2	OR52D1
1-Octen-3-ol		−4.29	−4.69	−4.13	−3.33	−3.44
1-Heptanol		−3.55	−4.06	−3.25	−3.34	−3.26
Benzeneacetaldehyde		−4.52	−3.26	−4.54	−3.26	−4.14
β-Ocimene		−5.12	−5.13	−5.11	−4.88	−4.43
Linalool		−5.2	−4.8	−5.2	−4.57	−4.01

**Table 6:** Summary of the interaction forces between olfactory receptors and ligands.

Receptor	Ligand	Hydrophobic Interactions	Hydrogen Bonds
OR1A1	1-Octen-3-ol	Met104, Ile105, Gly108, Asn109, Asp180, Phe206, Tyr258	Tyr178, Tyr276
	1-Heptanol	Phe73, Met104, Ile105, Gly108, Phe206, Asn209, Tyr250, Tyr258	Tyr178, Tyr276
	Benzeneacetaldehyde	Leu14, Gly16, Glu24, Met81, Asn84, His85, Phe177	-
	β-Ocimene	Phe73, Met104, Ile105, Asn109, Tyr178, Val203, Phe206, Tyr250, Tyr258, Tyr276	-
	Linalool	Phe73, Met104, Ile105, Gly108, Val203, Phe206, Val254, Tyr258	Asn109, Asn155, Gly202
OR1G1	1-Octen-3-ol	Leu15, Gly16, Phe17, Gln24, Leu27, Met81, Ile85, Gln90, Phe177	Leu14, Asn84
	1-Heptanol	Leu14, Leu15, Gly16, Gln24, Met81, Asn84, Gln90, Phe177	Phe17, Ser18
	Benzeneacetaldehyde	Leu14, Gly16, Phe17, Ser18, Gln24, Leu27, Met81, Ile85	Asn84
	β-Ocimene	Leu14, Leu15, Gly16, Phe17, Ser18, Gln24, Leu27, Asn84, Ile85, Gln88, Ser89, Gln90	-
	Linalool	Leu14, Gly16, Gln24, Asn84, Ile85, Gln88, Ser89, Gln90, Phe177	Met81
OR2W1	1-Octen-3-ol	Phe12, Thr163, Leu166, Thr168, Leu184	Tyr94, Leu164
	1-Heptanol	Leu174, His176, Glu180, Ala183, Lys186, Ile187, Lys268	Asp175, Ile173
	Benzeneacetaldehyde	Tyr104, Met105, Gly108, Ser109, Leu159, Ile206, Ile255	Asn155
	β-Ocimene	Phe73, Tyr104, Met105, Gly108, Asn155, Gly203, Phe251, Ile255, Tyr259	-
	Linalool	Phe73, Met105, Gly108, Gly203, Ile206, Val207, Ile255, Tyr259, Tyr278	Tyr104
OR1D2	1-Octen-3-ol	Leu199, Ile200, Gly203, Cys204, Phe207, Leu255, Tyr259	Tyr182
	1-Heptanol	Asp70, Phe73, Asp111, Leu115, Phe251, Tyr277, Thr281, Pro282	Ala247, Asn285
	Benzeneacetaldehyde	Leu101, Leu104, Val105, Val108, Tyr155, Glu180, Phe207, Tyr259	Met181
	β-Ocimene	Val105, Val108, Tyr155, Met181, Leu199, Gly203, Phe207, Tyr259	-
	Linalool	Tyr155, Leu199, Ile200, Gly203, Phe207, Leu255, Tyr259	Tyr182
OR52D1	1-Octen-3-ol	Glu183, Phe254, Pro257, Ala258, Ser261, Tyr284, Val285, Pro288	Tyr111, Tyr181
	1-Heptanol	Tyr111, Tyr181, Pro257, Ala258, Ser261, Ala281, Val285, Pro288	Phe254, Tyr284
	Benzeneacetaldehyde	Asn9, His10, Leu11, Arg167, Arg168, Leu169, Pro170	Tyr171
	β-Ocimene	Ala206, Leu207, Met210, Gly211, Tyr255, Ala258, Phe259, Phe262	-
	Linalool	Tyr111, Ala112, Phe162, Gly202, Val205, Ala206, Ala209, Met210	His108

## Data Availability

The original contributions presented in this study are included in the article. Further inquiries can be directed to the corresponding author.
